# SARS-CoV-2 spike protein induces IL-18-mediated cardiopulmonary inflammation via reduced mitophagy

**DOI:** 10.1038/s41392-023-01368-w

**Published:** 2023-03-09

**Authors:** Shuxin Liang, Changlei Bao, Zi Yang, Shiyun Liu, Yanan Sun, Weitao Cao, Ting Wang, Tae-Hwi Schwantes-An, John S. Choy, Samisubbu Naidu, Ang Luo, Wenguang Yin, Stephen M. Black, Jian Wang, Pixin Ran, Ankit A. Desai, Haiyang Tang

**Affiliations:** 1 State Key Laboratory of Respiratory Disease, National Clinical Research Center for Respiratory Disease, Guangdong Key Laboratory of Vascular Disease, Guangzhou Institute of Respiratory Health, The First Affiliated Hospital of Guangzhou Medical University, Guangzhou, Guangdong China; 2 College of Veterinary Medicine, Northwest A&F University, Yangling, Shaanxi China; 3 Department of Cellular Biology & Pharmacology, Herbert Wertheim College of Medicine, Miami, FL USA; 4 Department of Environmental Health Sciences, Robert Stempel College of Public Health and Social Work and Center for Translational Science, Florida International University, Port St. Lucie, FL USA; 5 Department of Medical and Molecular Genetics, Indiana University School of Medicine, Indianapolis, IN USA; 6 Department of Biology, The Catholic University of America, Washington, DC USA; 7 Krannert Institute of Cardiology, Department of Medicine, Indiana University, Indianapolis, IN USA; 8 Guangzhou Laboratory, Guangzhou, China

**Keywords:** Inflammation, Infectious diseases

## Abstract

Cardiopulmonary complications are major drivers of mortality caused by the SARS-CoV-2 virus. Interleukin-18, an inflammasome-induced cytokine, has emerged as a novel mediator of cardiopulmonary pathologies but its regulation via SARS-CoV-2 signaling remains unknown. Based on a screening panel, IL-18 was identified amongst 19 cytokines to stratify mortality and hospitalization burden in patients hospitalized with COVID-19. Supporting clinical data, administration of SARS-CoV-2 Spike 1 (S1) glycoprotein or receptor-binding domain (RBD) proteins into human angiotensin-converting enzyme 2 (hACE2) transgenic mice induced cardiac fibrosis and dysfunction associated with higher NF-κB phosphorylation (pNF-κB) and cardiopulmonary-derived IL-18 and NLRP3 expression. IL-18 inhibition via IL-18BP resulted in decreased cardiac pNF-κB and improved cardiac fibrosis and dysfunction in S1- or RBD-exposed hACE2 mice. Through in vivo and in vitro work, both S1 and RBD proteins induced NLRP3 inflammasome and IL-18 expression by inhibiting mitophagy and increasing mitochondrial reactive oxygenation species. Enhancing mitophagy prevented Spike protein-mediated IL-18 expression. Moreover, IL-18 inhibition reduced Spike protein-mediated pNF-κB and EC permeability. Overall, the link between reduced mitophagy and inflammasome activation represents a novel mechanism during COVID-19 pathogenesis and suggests IL-18 and mitophagy as potential therapeutic targets.

## Introduction

Coronavirus Disease 2019 (COVID-19) is caused by the severe acute respiratory syndrome coronavirus 2 (SARS-CoV-2). Presentation can range from mild symptoms to acute respiratory distress syndrome (ARDS) and fulminant myocarditis.^1^ While inflammation significantly contributes to disease severity and death,^2,3^ the precise molecular mechanisms of COVID-19 infection and cardiopulmonary complications remain unclear. Previously, activation of the NLR family pyrin domain containing 3 (NLRP3) inflammasome, a major driver of inflammation, has been observed in the lungs, sera and peripheral blood mononuclear cells (PBMCs) of patients with COVID-19. The NLRP3 inflammasome is a critical component of the immune system that mediates caspase-1 (Casp1) activation and the secretion of the pro-inflammatory cytokines, interleukin (IL)-1β and IL-18, in response to infection and cell injury. Increased NLRP3 as well as apoptosis-associated speck-like protein (ASC) expression and Casp1 activity were linked with heightened expression levels of mature IL-1β and IL-18 in COVID-19 samples.^4–6^ Although prior analyses were unadjusted, the magnitude of the inflammasome activation along with IL-1β and IL-18 levels were also proposed to correlate with disease severity and clinical outcomes in patients with COVID-19.^4,7^

IL-18, a pro-inflammatory cytokine of the IL-1 family, is synthesized as an inactive precursor, processed to its active form by Casp1, and released.^8^ Based on prior reports of its role in multiple forms of lung injury including ARDS,^9^ SARS^10^, and influenza,^11^ IL-18 is expected to contribute to inflammatory lung injury caused by SARS-CoV-2 infection. Lung samples from COVID-19 infected individuals demonstrate excessive neutrophil recruitment, infiltration, and activation, all established functions of IL-18.^12–15^ These observations support a critical role for IL-18 in COVID-19 lung injury. Beyond lung damage, IL-18 also mediates the development of cardiovascular dysfunction, mimicking the spectrum of cardiac pathology observed in patients with COVID-19. Endogenous IL-18 has been shown to contributes to contractile dysfunction following myocardial ischemia,^16^ while excess IL-18 mediates cardiac inflammation, fibrosis, and ventricular tachycardia.^17^ The regulation and role of IL-18 in COVID-related cardiopulmonary injury, however, remains unclear.

Aging is one of the strongest predictors of poor outcomes of COVID-19.^18,19^ Aging is associated with increased basal levels of inflammation and declining mitochondrial function.^20,21^ Mitochondrial dysfunction is known to regulate IL-18-dependent inflammasome signaling.^22^ Mitophagy, a selective autophagic process, initiates and targets impaired mitochondria for lysosomal degradation and plays a major role in maintaining mitochondrial quality control and homeostasis.^23^ Inhibition of mitophagy stimulates the production of mitochondrial reactive oxygenation species (ROS), leading to increased inflammasome activation.^22,24^ Given the association between SARS-CoV-2 infection and observations of mitochondrial dysfunction^25^ as well as the role of mitophagy and IL-18 signaling in other viral infections, such as influenza A virus,^26^ we hypothesized that alterations in mitophagy contributes to cardiopulmonary injury via SARS-CoV-2 signaling and IL-18 activation.

SARS-CoV-2 Spike (S) glycoprotein modulates viral entry into host cells by binding to the host receptor ACE2, through the receptor-binding domain (RBD) in the S1 subunit. Using both S1 and RBD proteins, we investigated the molecular mechanisms linking mitochondrial dysfunction to inflammasome-dependent IL-18 signaling, and the development of cardiopulmonary injury. We found that administration of Spike protein causes increased temperatures, NF-κB activity and NLRP3 inflammasome-dependent IL-18 levels in heart and lung tissues, and impairs cardiopulmonary function in hACE2-knock in (KI) mice. IL-18 inhibition improved cardiac function and reduced NF-κB activity in mice. Mechanistically, we demonstrate that Spike protein activates NLRP3 inflammasome by increasing mitochondrial ROS (mitoROS) generation and mitophagy blockade across three relevant lines including hACE2-expressing H9C2 cells, human pulmonary arterial endothelial cells (HPAEC) and cardiac microvascular EC (HCMEC). Furthermore, IL-18 inhibition via IL-18BP prevented Spike protein-mediated NF-κB phosphorylation and EC permeability. Taken together, these findings reveal a novel link between Spike signaling, mitophagy inhibition, and IL-18 activation, highlighting potential therapeutic targets for major COVID-19 cardiopulmonary complications.

## Results

### Hospitalization burden and survival curves stratified by IL-18 levels

**Fig. 1:**
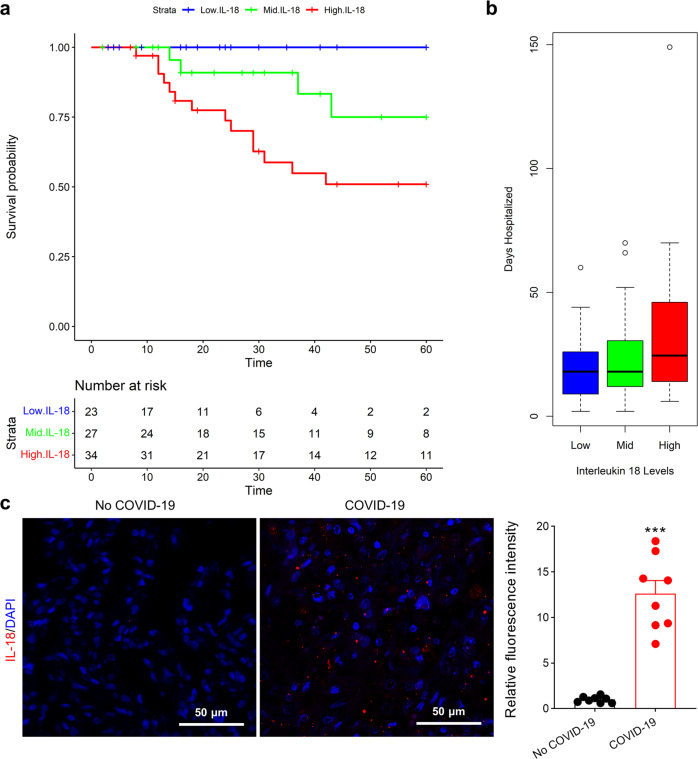
Hospitalization burden and survival curves stratified by IL-18 levels after COVID-19 diagnosis. **a** Kaplan–Meier curves illustrate significant (*P*-value = 5.71E−03) overall survival differences stratified by IL-18 tertiles based on time from COVID-19 test to 60-day follow-up (discharge or death). There was worse survival if IL-18 levels were high (red, above cutoffs of 102 pg/mL versus green, 48.6–102 pg/mL versus blue, below cutoffs of 48.6 pg/mL). Each line indicates the predicted survival probability over follow-up time. **b** Boxplot of IL-18 tertiles and days spent hospitalized is plotted. **c** IL-18 fluorescence microscopy of human lung from COVID-19 patients vs. non-COVID-19 patients

Analysis of demographic data and levels of 19 cytokines from 109 individuals diagnosed with COVID-19 revealed that IL-18 was among the top three cytokines that was differentially regulated and significantly elevated in those who died compared to those who survived infection (Table 1). As expected, age remained a significant risk factor for poor outcomes in this cohort (mean age in survivors was 58 years and 77 years among those that died). After multi-variate adjustment for sex, age, and race, cox proportional hazard ratios were calculated for all of the inflammatory cytokines (Table 2). IL-18 remained one of the top three significant cytokines with the highest hazard ratios. We also tested the association between age and IL-18 level by regression. We observed a significant association of age on IL-18, (*p*-value 0.0002, beta = 0.02 unit increase per year of age), suggesting that IL-18 levels are associated with increasing age. In mediation analysis with vital status, however, age became non-significant (*p* = 0.3, beta 0.008), suggesting that the effect of age on IL-18 levels was mediated by outcome status. Consistent with prior reports,^27^ IL-6, well-known to associate with poor survival during COVID-19, IL-27, and IL-15 were also observed to stratify survival, but IL-6 displayed a lower hazard ratio than IL-18. Kaplan–Meier curves illustrate significant overall survival differences based on IL-18 levels starting from time of COVID-19 test to both 60-day follow-up (discharge or death, Fig. 1a) as well as to last follow-up (Supplementary Fig. 1a). After adjustment for sex, age, and race, IL-18 levels were also significantly associated with risk of hospitalization day burden [*P*-value = 0.03, beta (β) = 5.68 days per natural log unit 1 increase in log (IL-18) levels] defined as total number of days hospitalized after COVID-19 diagnosis (Fig. 1b). These data suggest IL-18 amongst the top cytokines that stratify morbidity and mortality from COVID-19. Further supporting these associations from circulating blood levels, Patients with COVID-19 exhibited elevated IL-18 (Fig. 1c and Supplementary Fig. 2) and IL-15 expression (Supplementary Fig. 3) in their lung tissues compared with control patients without COVID-19.

**Table 1:** Demographic and cytokine data of IU COVID-19 cohort

	Total (*N* = 109)	Alive (*N* = 88)	Dead (*N* = 21)	*P* value
Age (yrs)	62.2 (16.3)	58.3 (15.5)	77.4 (9.0)	1.07E−09
Sex (% male, *n*)	56% (59)	56% (47)	57% (12)	1.00
Race (% non-white, *n*)	49% (51)	46% (39)	62% (13)	0.23
Total hospitalized days	20 (12–35)	21 (11–37)	19 (12–26)	0.94
IL-1a	21.22 (32.68)	25.02 (36.31)	8.14 (3.67)	0.02
IL-1b	25.56 (51.31)	23.31 (38.12)	34.24 (86.83)	0.65
IL-1RA	103.8 (285.88)	80.89 (244.08)	198.7 (411.49)	0.22
IL-5	6.79 (7.25)	6.6 (7.18)	7.57 (7.67)	0.60
IL-6	190.33 (909.66)	171.73 (990.34)	266.51 (458.01)	0.52
IL-7	14.39 (91.63)	17.03 (104.49)	5.65 (10.43)	0.44
IL-8	8.78 (14.62)	6.83 (9.54)	16.94 (25.9)	0.09
IL-9	15 (15.18)	15.62 (16.69)	12.75 (7.37)	0.37
IL-10	59.12 (224.1)	57.87 (253.83)	62.56 (110.51)	0.91
IL-12	81.5 (70.86)	77.92 (51.9)	96.5 (123.12)	0.52
IL-13	59.58 (148.77)	63.85 (162.63)	38.24 (23.65)	0.32
IL-15	16.8 (11.67)	15.54 (11.37)	22.01 (11.74)	0.03
IL-17	187.61 (621.7)	217.7 (691.21)	61.24 (46.25)	0.32
IL-18	111.5 (153.8)	80.1 (82.8)	239.8 (273.4)	0.02
IL-27	3290.92 (3292.31)	2488.37 (1979.54)	5564 (5174.59)	0.0015

^*^Presented as mean pg/mL (standard deviation)

**Table 2:** Hazard ratios of circulating cytokines from IU COVID-19 cohort

Interleukin (log)	HR	L95-U95	Sample size	Deaths	*P* value
**IL-27**	2.80	1.3–6.03	86	20	**0.01**
**IL-15**	2.75	1.13–6.7	85	20	**0.03**
**IL-18**	2.62	1.33–5.15	84	20	**0.01**
**IL-6**	1.77	1.27–2.46	86	20	**<0.01**
IL-1a	0.26	0.02–3.51	28	8	0.31
IL-1b	1.00	0.5–2	54	13	1.00
IL-1RA	1.23	0.9–1.67	85	20	0.19
IL-5	0.68	0.4–1.18	86	20	0.17
IL-7	1.44	0.71–2.92	56	15	0.31
IL-8	1.43	0.79–2.57	86	20	0.24
IL-9	1.74	0.5–6.07	48	12	0.39
IL-10	1.55	0.96–2.51	65	20	0.07
IL-12	0.99	0.5–1.97	81	19	0.97
IL-13	0.99	0.29–3.34	40	8	0.99
IL-17	0.81	0.29–2.31	41	10	0.70

^*^HR = Cox proportional hazard ratio (adjusted for age, sex, race); L95-U95 = upper and lower 95% confidence intervals

Bold values show *p* value < 0.05 in these cytokines

### SARS-CoV-2 Spike protein induces murine cardiopulmonary dysfunction

**Fig. 2:**
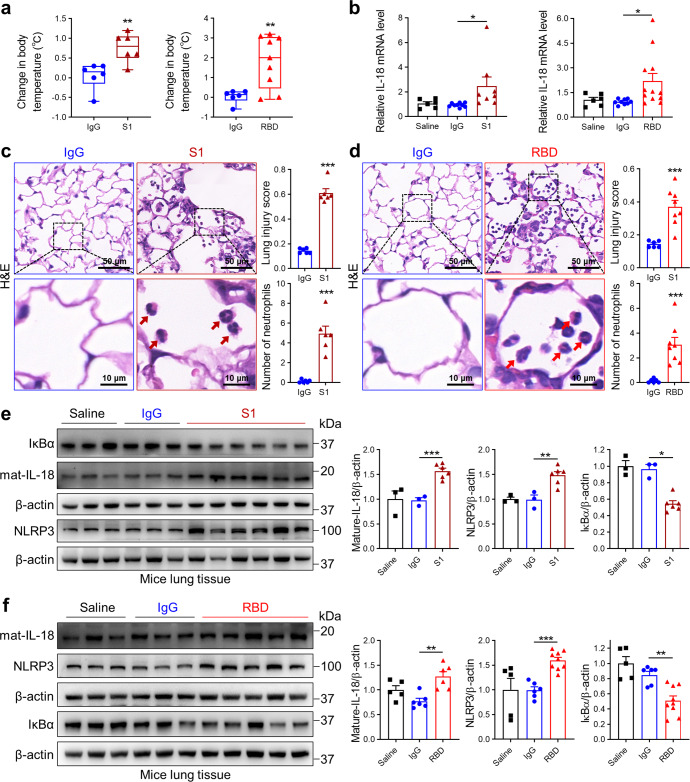
Spike protein induces lung inflammation and IL-18 expression. Transgenic hACE2 mice were administered recombinant SARS-CoV-2 Spike S1 and RBD protein (5 μg/mouse/d) for via tracheal intubation. After 10 days, mice were sacrificed. **a** Body temperature change is more in Spike protein-treated mice compared to IgG-treated mice (Student’s *t*-test, *N* = 6–9). **b** IL-18 gene expression levels are higher in the blood of Spike protein-treated mice (Student’s *t*-test). **c**, **d** Lung sections were examined by hematoxylin and eosin staining. Representative micrographs, neutrophil cell count and scored for lung injury are shown (Student’s *t*-test, *N* = 6–8). Red arrows suggest neutrophils. **e**, **f** Representative western blots and quantifications of IκBα (Student’s *t*-test), IL-18 (Student’s *t*-test) and NLRP3 (Student’s *t*-test) in lung tissues from saline-, IgG- and RBD/S1 protein-treated mice (*N* = 3–9). Data indicate mean ± SE. *p < 0.05, **p < 0.01, ***p < 0.001 relative to IgG exposure

Spike protein of SARS-CoV-2 is processed proteolytically into the S1 receptor-binding subunit and the S2 membrane fusion subunit, and SARS-CoV-2 RBD mediates virus entry into host cells via binding to ACE2.^28^ To better understand the association between IL-18 levels and morbidity and mortality in patients with COVID-19, we studied Spike signaling via S1 and RBD protein signaling in a pre-clinical mouse model. The hACE2 transgenic mouse model recapitulates many of the manifestations observed in patients with COVID-19 including fever and inflammatory lung injury.^29^ We also observed increased body temperatures (Fig. 2a) with daily S1 or RBD tracheal administration in these mice. Moreover, IL-18 mRNA levels were elevated in the blood (Fig. 2b) after S1 and RBD exposure. Inflammatory lung injury was apparent in transgenic mice receiving Spike protein, validated by increased infiltration of neutrophils into the lungs and signs of lung tissue damage (Fig. 2c, d). S1 and RBD protein administration also increased levels of NLRP3 and IL-18 (Fig. 2e, f) in lung tissue. Given the established role of NF-κB activation in the inflammasome,^30^ we next evaluated NF-κB and IκBα levels and found that S1 and RBD protein administration were also associated with decreased IκBα levels (Fig. 2e, f).

**Fig. 3:**
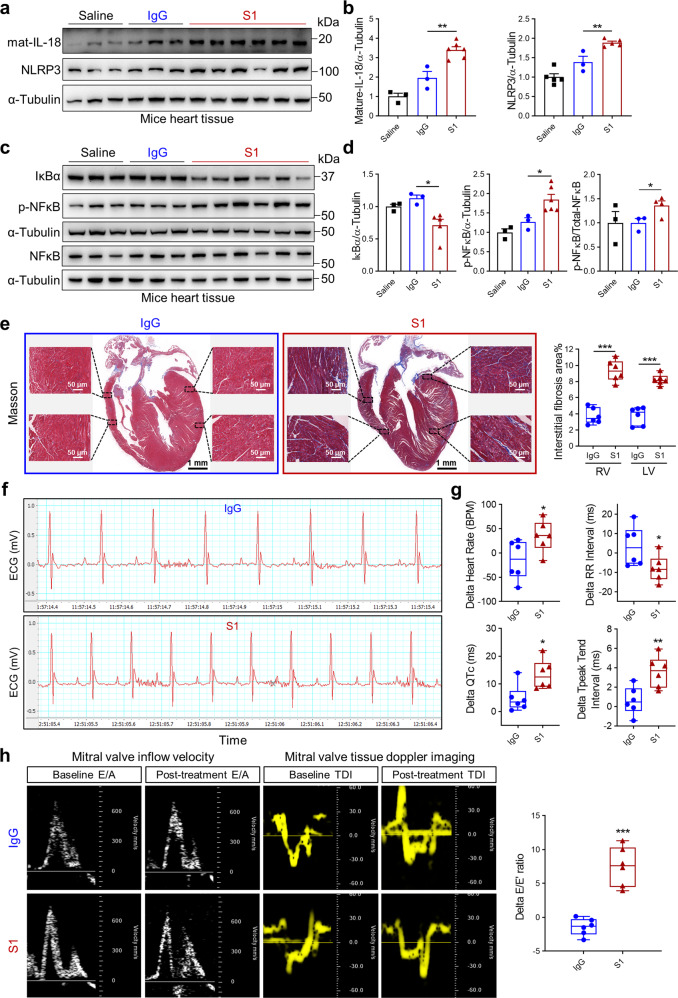
Spike 1 protein induces cardiac IL-18 expression, fibrosis, and heart dysfunction in hACE2-KI mice. **a**, **b** Western blots of IL-18 and NLRP3 levels in heart tissues. Quantitative data shown in right panel (**b**) (Student’s *t*-test, *N* = 3–5). Representative western blots showing protein levels of IκBα, NF-κB, phospho-NF-κB (**c**) and summarized data (**d**) in heart tissues (Student’s *t*-test, *N* = 3–6). **e** Representative Masson Trichrome staining images of heart from IgG- versus S1 protein-treated mice. Fibrosis area percentage was higher in S1 protein-treated mice heart tissues (Student’s *t*-test, *N* = 6). **f**, **g** Heart rate, RR interval, QTc interval, Tp-Te interval were measured by electrocardiograms (ECG) in mice after S1 exposure (Student’s *t*-test, *N* = 6). **h** Representative echocardiogram images of mitral valve inflow velocity and tissue doppler and summarized data showing impaired diastolic function (increased delta *E*/*E*’) after S1 exposure (Student’s *t*-test, *N* = 6). Values are mean ± SE. *p < 0.05, **p < 0.01, ***p < 0.001 relative to IgG exposure

**Fig. 4:**
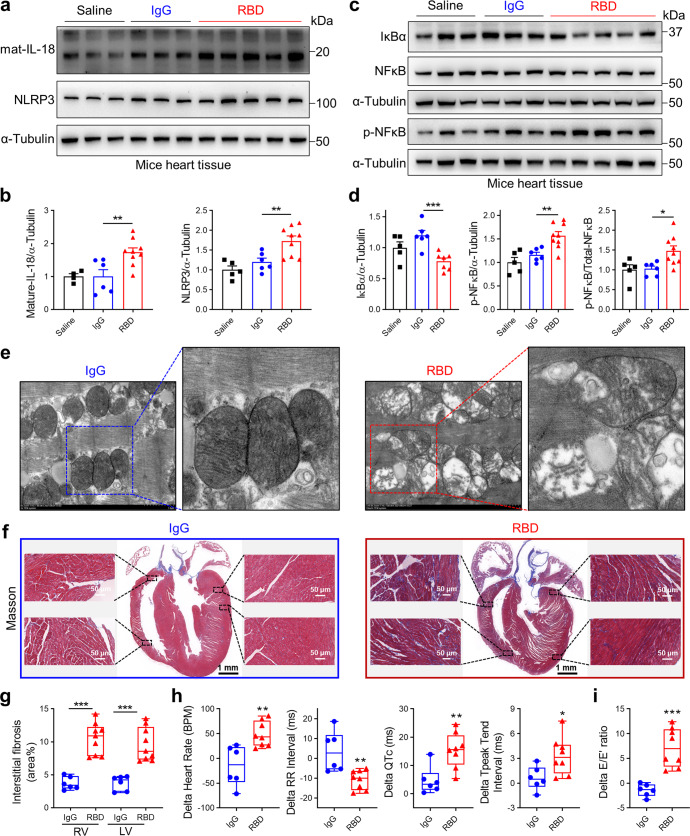
Spike RBD protein induces cardiac IL-18 expression, fibrosis mitochondria injury, heart dysfunction in hACE2-KI mice. **a**, **b** Western blots of IL-18 and NLRP3 levels in heart tissues. Quantitative data shown in panel below (**b**) (Student’s *t*-test, *N* = 5–9). Representative western blots showing IκBα, NF-κB, phospho-NF-κB protein levels (**c**) and summarized data (**d**) in heart tissues (Student’s *t*-test, *N* = 5–9). **e** Representative images from transmission electron microscopy (TEM) after IgG and RBD treatment. **f**, **g** Representative Masson Trichrome staining images of heart from IgG- versus RBD protein-treated mice. Fibrosis area percentage was higher in RBD protein-treated mice heart tissues (Student’s *t*-test, *N* = 6–9). **h** Heart rate, RR interval, QTc interval, Tp-Te interval were measured by electrocardiograms (ECG) in mice after RBD exposure (Student’s *t*-test, *N* = 6–8). **i** Diastolic function was impaired (increased delta *E*/*E*’) after RBD exposure (Student’s *t*-test, *N* = 6–8). Values are mean ± SE. *p < 0.05, **p < 0.01, ***p < 0.001

Furthermore, orotracheal administration of Spike protein in hACE2 transgenic mice increased NLRP3 and IL-18 protein levels (Figs. 3a, b and 4a, b) in cardiac tissues. Levels of IκBα were decreased while phosphorylated NF-κB levels and ratio of phosphorylated NF-κB to total NF-κB were increased in heart tissues of Spike protein-treated mice (Figs. 3c, d and 4c, d). These results suggest that S1 and RBD protein activate NLRP3 inflammasome-induced IL-18 signaling associated with NF-κB activation and cardiopulmonary injury. Abnormal mitochondrial morphology has previously been detected during SARS-CoV-2 infection.^31,32^ Supporting these prior observations, transmission electron microscopy (TEM) images (Fig. 4e) revealed increased irregular mitochondrial arrangement and reduced density in the hearts of RBD protein-treated mice, suggesting Spike protein mediates impaired mitochondrial function.

Consistent with reports of cardiac injury in COVID-19 patients,^33,34^ exposure to S1 and RBD protein resulted in increased myocardial collagen deposition and fibrosis (Figs. 3e and 4f, g). Further, ECG monitoring displayed significantly higher heart rate, longer QTc, Tp-Te and lower RR intervals after S1 and RBD protein treatment, suggesting increased risk factors for arrhythmias (Figs. 3f, g and 4h). Echocardiography detected increased E/E’ after S1 and RBD protein administration (Figs. 3h and 4i), reflecting increased LV filling pressures. These data cumulatively suggest that Spike protein mediates the development of cardiopulmonary injury and further fuel the notion that the cleaved form of the Spike protein, S1, alone can elicit a strong inflammatory response.^35^

### IL-18 inhibition attenuates Spike-mediated cardiac dysfunction in mice

**Fig. 5:**
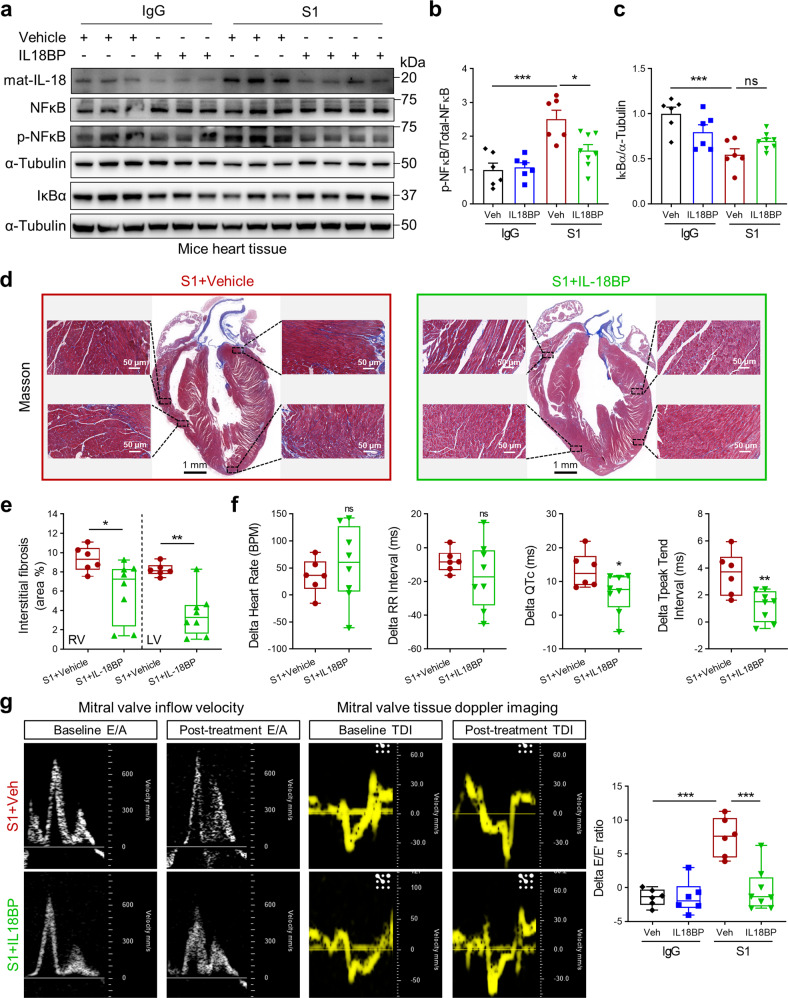
IL-18BP reduces fibrosis and improves function in Spike protein-treated hACE2-KI mice. **a**–**c** Western blots of IL-18, NF-κB, phospho-NF-κB and IκBα levels in heart tissues. Quantitative data shown in right panel (**b**, **c**) (1-way ANOVA test, *N* = 5–8). **d**, **e** Representative Masson Trichrome staining images of heart from IgG- versus S1 protein-treated mice with IL-18BP treatment. Fibrosis area percentage was reduced with IL-18BP injection (Student’s *t*-test, *N* = 6–8). **f** Heart rate, RR interval, QTc interval, Tp-Te interval were measured by electrocardiograms (ECG) in mice after S1 and IL-18BP exposure (Student’s *t*-test, *N* = 6–8). **g** IL-18BP exposure improved impaired diastolic function (1-way ANOVA test, *N* = 6–8). Values are mean ± SE. *p < 0.05, **p < 0.01, ***p < 0.001

To determine whether IL-18 may represent a novel therapeutic target for Spike-mediated cardiopulmonary injury, we administered its decoy receptor, IL-18BP, after exposing mice with Spike protein for 5 days. After injecting IL-18BP for an additional 5 days in both the vehicle and Spike protein-treated hACE2 transgenic mice, IL-18 levels were measured in the heart tissues and in serum. Administration of IL-18BP significantly reduced Spike protein-mediated activation of NF-κB (Fig. 5a, b). IκB levels were not different after IL-18BP injections (Fig. 5a, c). IL-18 inhibition also reduced both cardiac fibrosis as measured by interstitial fibrosis area percentage (Fig. 5d, e) and cardiac dysfunction (Fig. 5g). In addition, IL-18BP-exposed mice displayed reduced changes in QTc and Tp-Te intervals (Fig. 5f).

### Spike protein induces IL-18 expression via reduced mitophagy and increased mitoROS production in vivo

**Fig. 6:**
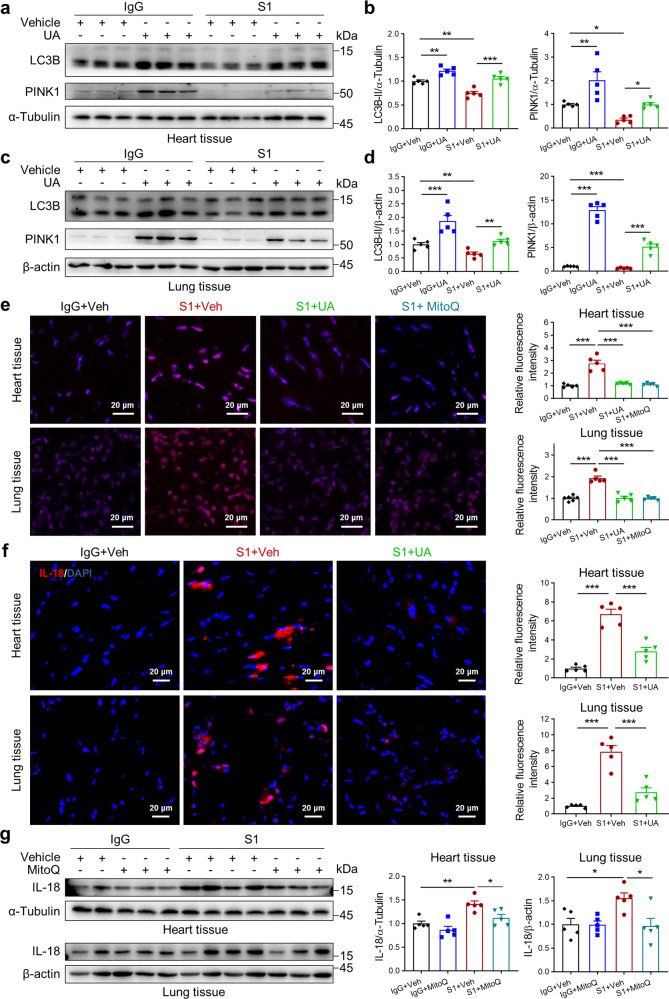
Spike protein induces IL-18 expression via reduced mitophagy and increased mitoROS production in vivo. Western blots (**a**) and quantitative data (**b**) of LC3B and PINK1 levels in heart tissues from IgG- versus S1 protein-treated mice with UA treatment (1-way ANOVA test, *N* = 5). Western blots (**c**) and quantitative data (**d**) of LC3B and PINK1 levels in lung tissues from IgG- versus S1 protein-treated mice with UA treatment (1-way ANOVA test, *N* = 5). **e** Representative DHE staining images and quantitative data of heart and lung from IgG- versus S1 protein-treated mice with UA or MitoQ treatment (1-way ANOVA test, *N* = 5). Scale bar, 20 μm. **f** Representative immunofluorescence images and quantitative data showing stained IL-18 in heart and lung tissue from IgG- versus S1 protein-treated mice with UA treatment (1-way ANOVA test, *N* = 5). Nuclei counterstained with DAPI. Scale bar, 20 μm. **g** Western blots and quantitative data of IL-18 levels in heart and lung tissues from IgG- versus S1 protein-treated mice with MitoQ treatment (1-way ANOVA test, *N* = 5). Values are mean ± SE. *p < 0.05, **p < 0.01, ***p < 0.001

Considering the roles of mitophagy and mitoROS in IL-18-dependent inflammasome signaling, urolithin A (UA, a mitophagy inducer) and mitochondrion-targeted antioxidant mitoquinone (MitoQ) were utilized to examine whether both were involved during in vivo modeling in S1 protein-mediated inflammasome activation. As shown in Fig. 6a–d, S1 protein treatment reduced mitochondrial membrane kinase, PINK1 (PTEN-induced putative kinase 1) and autophagosome marker, LC3B-II levels in heart and lung tissues of hACE2 transgenic mice. UA administration in these experiments, however, recovered levels of PINK1 and LC3B-II (Fig. 6a–d). Moreover, Dihydroethidium (DHE) and immunofluorescent staining revealed that UA treatment also attenuated the S1 protein-mediated IL-18 upregulation by inhibiting ROS production (Fig. 6e, f). To further confirm the role of mitoROS in S1 protein-mediated IL-18 activation, we also quantified changes in IL-18 protein levels after MitoQ administration. As presented in Fig. 6e, g, MitoQ attenuated S1 protein-mediated IL-18 upregulation via reducing ROS production in heart and lung tissues of hACE2 transgenic mice. These results demonstrate that Spike protein induces IL-18 production, in part, via the inhibition of mitophagy and activation of mitoROS.

### Spike protein induces IL-18 expression via reduced mitophagy and increased mitoROS production in hACE2-expressing H9C2 cells

**Fig. 7:**
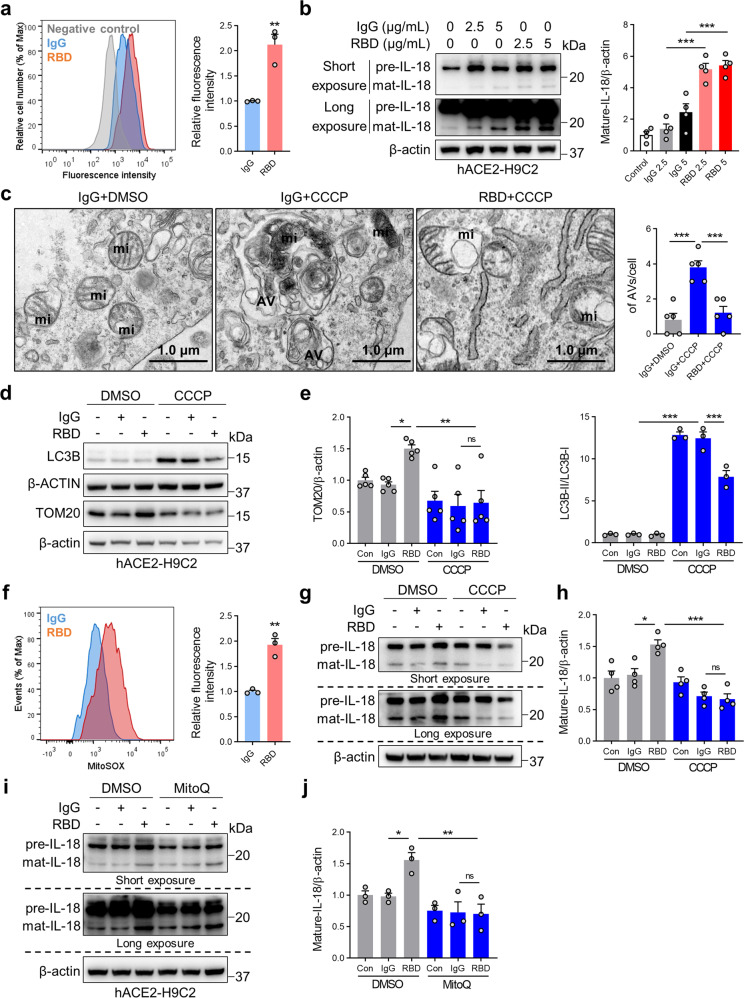
Spike protein augments IL-18 levels in H9C2 cells via impairment of mitophagy and mitochondrial ROS production. **a** After incubation with RBD protein (2.5 μg/mL) for 3 h, binding of RBD protein to human ACE2 was detected by FACS. Representative histograms (left panel) and summarized data (right panel) are presented. The *X*-axis represents fluorescence intensity, and the *Y*-axis shows cell number normalized as a percentage of the maximum (% of max) (Student’s *t*-test, *N* = 3). **b** IL-18 protein levels were measured using western blot (1-way ANOVA test, *N* = 4). **c** Representative TEM images of mitochondrial structure in hACE2-H9C2 cells treated with IgG (2.5 μg/mL) or RBD (2.5 μg/mL) protein in the presence of CCCP (10 μM) (1-way ANOVA test, *N* = 5). Representative immunoblot (**d**) and quantitative analysis (**e**) of LC3B and TOM20 in IgG (2.5 μg/mL) or RBD (2.5 μg/mL), -treated hACE2-H9C2 cells with or without CCCP (10 μM) (1-way ANOVA test, *N* = 5). **f** Flow cytometry of hACE2-H9C2 cells labeled with MitoSOX Red after treatment with RBD protein. Data are representative of three independent experiments (Student’s *t*-test, *N* = 3). **g**, **h** Western blot analysis of IL-18 in the presence of CCCP (10 μM) and RBD protein (2.5 μg/mL) (1-way ANOVA test, *N* = 4). **i**, **j** hACE2-H9C2 cells were treated with IgG (2.5 μg/mL) or RBD (2.5 μg/mL) protein in the presence of MitoQ (1 μM) or DMSO incubated for 1 h. Samples were immunoblotted with IL-18 antibody (1-way ANOVA test, *N* = 3). Data are shown as the mean ± SE. *p < 0.05, **p < 0.01, ***p < 0.001, ns no significant differences. AV autophagic vacuole; mi mitochondrion

To further confirm the mechanisms of Spike protein-induced NLRP3 inflammasome and cardiac IL-18 activation, hACE2-expressing lentivirus were transduced to H9C2 cells followed by exposure to RBD protein. Increased ACE2 expression in cells was confirmed by western blotting (Supplementary Fig. 4a). Flow cytometry was used to confirm RBD protein binding (Fig. 7a). Expression of NLRP3 inflammasome mediators and IL-18 were markedly upregulated in RBD protein-treated cells consistent with NLRP3 activation (Fig. 7b and Supplementary Fig. 4b, c). Notably, IL18BP, a native protein which binds to IL-18 and prevents it from activating its receptor, inhibited Spike protein-induced NF-κB activation (Supplementary Fig. 4d, e), indicating that IL-18 may mediate cardiac inflammation via NF-κB activation.

Consistent with in vivo observations, TEM confirmed the presence of mitochondrial swelling, severe disrupted cristae, and vacuole formation in RBD protein-treated cells (Fig. 7c). Since mitophagy plays a key role in mitochondrial quality control and homeostasis,^23,36^ a mitochondrial toxin, carbonyl cyanide *m*-chlorophenyl hydrazone (CCCP), which inhibits oxidative phosphorylation, was used to induce mitophagy. TEM showed that the autophagic vacuole containing mitochondria existed in CCCP-treated cells, while RBD protein inhibited their formation (Fig. 7c). Immunoblots also revealed that RBD protein exposure increased the levels of mitochondrial marker, TOM20 and inhibited CCCP-mediated increases in autophagosome marker, LC3B-II levels, indicating an impairment of mitophagy in the presence of RBD protein (Fig. 7d, e). Flow cytometry analysis confirmed increased mitoROS production (Fig. 7f) with RBD protein exposure while increased NOX4 levels were also observed on immunoblots (supplementary Fig. 4f, g). These data demonstrate increased mitochondrial injury and mitoROS production and decreased mitophagy with RBD protein-treated cells.

Immunoblot analysis showed that cells treated with CCCP prevented RBD protein-induced activation of NLRP3 inflammasome, evidenced by decreased NLRP3, ASC, and mature IL-18 levels (Fig. 7g, h; Supplementary Fig. 4h, i). Furthermore, cells treated with MitoQ also had significantly reduced levels of mature IL-18 in RBD protein-treated cells (Fig. 7i, j). These data link RBD protein-mediated attenuation of mitophagy to the increased generation of mitoROS production required for NLRP3 inflammasome-mediated IL-18 activation in hACE2-H9C2 cells.

### Spike protein increases EC permeability and induces IL-18 expression via inhibition of mitophagy

**Fig. 8:**
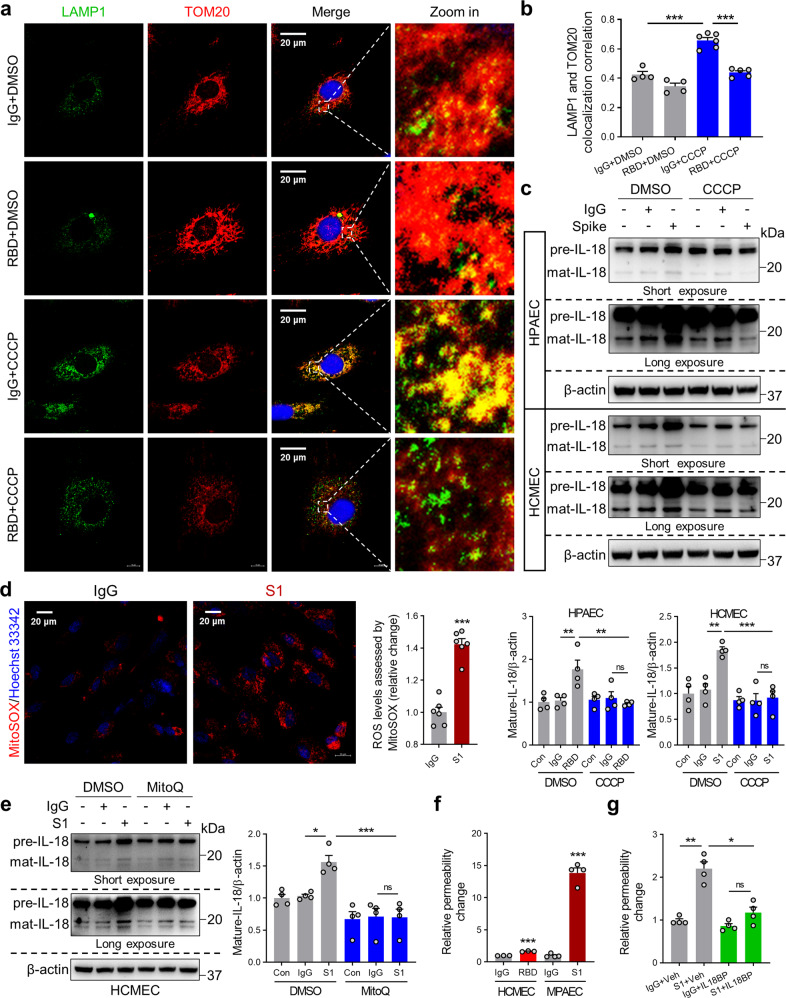
Spike protein induces IL-18 expression via impairment of mitophagy resulting in EC permeability. Representative immunofluorescence images (**a**) and summary data (**b**) showing stained TOM20 and LAMP1 in HPAEC in the presence of CCCP (10 μM) and RBD protein (2.5 μg/mL) (1-way ANOVA test, *N* = 4–6). Nuclei counterstained with DAPI. Scale bar, 20 μm. **c** Protein extracts were subjected to western blot analysis for IL-18 after CCCP and RBD/S1 (2.5 μg/mL) protein treatment in HPAEC and HCMEC (1-way ANOVA test, *N* = 4). **d** Representative images of MitoROX staining and quantitative analysis of fluorescence intensity in IgG- and S1 protein-treated HCMEC. Scale bar, 20 μm (Student’s *t*-test, *N* = 6). **e** Representative immunoblots and quantitative analysis of IL-18 in HCMEC with or without IgG (2.5 μg/mL) or S1 (2.5 μg/mL) protein and MitoQ (1 μM) stimulation (1-way ANOVA test, *N* = 4). **f** Measurement of endothelial cell permeability in HCMEC or mice primary PAEC (MPAEC) treated with Spike protein (Student’s *t*-test, *N* = 3–4). **g** Measurement of permeability in HPAEC treated with S1 protein (5 μg/mL) and IL-18BP (2 μg/mL) (1-way ANOVA test, *N* = 4). Results represent means ± SE. *p < 0.05, **p < 0.01, ***p < 0.001. ns no significant differences

While EC dysfunction has been observed in COVID-19 patients,^37–39^ the underlying mechanisms of endothelial cell damage and inflammation remain unclear. Using flow cytometry, we found that RBD protein binds to both HPAEC and HCMEC (Supplementary Fig. 5a, b). Mirroring H9C2 cells, S1 and RBD protein-treated ECs showed similar increases in NLRP3, ASC, and mature IL-18 levels (Supplementary Fig. 5c). IL-18BP treatment inhibited Spike protein-associated NF-κB activation (Supplementary Fig. 5d). Moreover, immunoblots revealed RBD protein attenuated CCCP-induced mitophagy (Supplementary Fig. 5e). These latter data were supported by co-staining with TOM20 (mitochondrial marker) and LAMP1 (lysosome marker). We observed RBD protein attenuated CCCP-stimulated colocalization of mitochondria and lysosomes (Fig. 8a, b). CCCP treatment attenuated Spike protein-mediated increases in NLRP3, ASC and mature IL-18 expression (Fig. 8c; Supplementary Fig. 5f). Similarly, S1 protein also induced mitoROS production in HCMEC (Fig. 8d). MitoQ treatment also inhibited IL-18 expression (Fig. 8e). Considering the established role of ACE2/AngII/Ang-(1–7) axis in SARS-CoV-2-induced NLRP3 inflammasome activation,^40^ IL-18 levels were examined in the presence of Ang-(1–7). As shown in Supplementary Fig. 6a, b, Ang-(1–7) attenuated activation of IL-18 in hACE2-H9C2 cells and in HPAEC, further supporting the role of ACE2/ AngII/Ang-(1–7) axis in SARS-CoV-2-induced NLRP3 inflammasome.

Given their crucial role in maintaining vascular permeability, homeostasis, and blood rheology, EC dysfunction directly contributes to cardiopulmonary inflammation during COVID-19.^41^ We, therefore, modeled EC permeability in the presence or absence of Spike protein to determine the role for IL-18. Vascular permeability assay demonstrated that Spike protein exposure resulted in increases in HPAEC and mice primary PAEC permeability (Fig. 8f). Treatment with IL-18BP reduced Spike protein-induced the increase of permeability (Fig. 8g).

## Discussion

**Fig. 9:**
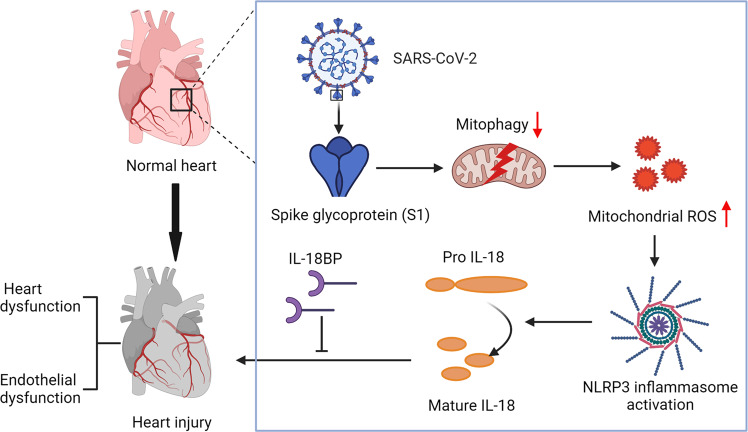
Model illustrating the associations between SARS-CoV-2 signaling, mitophagy inhibition, IL-18 activation, and heart injury. Upon infection, Spike protein can inhibit mitophagy and induces mitochondrial ROS production, thus leading to inflammasome activation, IL-18 maturation, and the occurrence of heart injury. The schematic illustration was designed by BioRender

Cardiopulmonary injury and inflammation are major drivers of morbidity and mortality in patients with COVID-19. Older populations are especially vulnerable to poor cardiopulmonary outcomes with COVID-19. Accumulation of damaged mitochondria is commonly found during ageing, inflammation and cardiopulmonary dysfunction. In fact, reduced mitophagy is observed in a variety of cardiopulmonary diseases and results in induction of inflammasome signaling.^24^ However, the role of mitophagy in COVID-19-mediated inflammation remains unknown. For the first time, the current work investigates viral-mediated cellular mechanisms and demonstrates a link between inflammation and mitophagy in both cardiac and pulmonary tissues. We were able to prioritize IL-18 among 19 cytokines to predict hospitalization burden and mortality. Increased IL-18 levels also exist in lung tissues of COVID-19 patients and SARS-CoV-2-infected mice. Further, we found that S1 and RBD both induced IL-18 activation in heart and lung, endothelial cells and, in vivo, in hACE2 transgenic mice. Mechanistically, we demonstrate that Spike proteins induce IL-18 expression by increasing mitoROS generation and mitophagy blockade in three cardiopulmonary relevant cell lines and in vivo, in mice. Blockade of IL-18 via IL-18BP attenuated Spike-mediated cardiopulmonary inflammation and injury. Taken together, these findings support a novel link between SARS-CoV-2 signaling, mitophagy inhibition, and IL-18 activation, highlighting novel therapeutic targets for major COVID-19 cardiopulmonary complications (Fig. 9).

Strict biosafety requirements continue to limit working with live coronavirus. Thus, several virus-free mice models have been developed for mechanistic COVID-19 studies.^42,43^ The current study utilized recombinant SARS-CoV-2 Spike protein in hACE2 transgenic mice to mimic SARS-CoV-2 Spike signaling in vivo. Our data re-produce previously published reports demonstrating evidence of fever, lung and heart inflammation as well as neutrophil infiltration.^42,44^ Importantly, COVID-19-associated cardiac manifestations were also observed including myocardial fibrosis, changes in electrical intervals, and diastolic dysfunction in these mice. These phenotypes bolster the use of this mouse model to study the molecular mechanisms of cardiopulmonary injury during COVID-19 pathogenesis. Moreover, findings from experiments involving RBD-mediated cardiopulmonary injury suggest that blockade of the interaction between SARS-CoV-2 RBD protein and hACE2 may represent a novel treatment strategy for COVID-19 beyond the current focus weighted toward S1 protein.

COVID-19 pathogenesis and outcomes are directly correlated with the presence of a hyper-inflammatory response, including inflammasome activation.^4^ There is also evidence of cardiopulmonary injury including acute lung injury, myocardial injury, myocarditis, and arrhythmia.^45^ IL-18 represents a well-established marker of inflammasome activation and has been linked to disease severity and mortality caused by cytokine storm in acute lung injury.^46^ Beyond lungs, IL-18 is also a mediator of cardiac fibrosis, dysfunction, and arrhythmias.^17^ In line with these prior observations, our data demonstrate that cardiac fibrosis and dysfunction, as well as lung inflammation are accompanied by an increase in IL-18 activation in hACE2 transgenic mice after exposure to different components of the Spike protein. Based on known associations between inflammasome activation and cardiovascular and pulmonary outcomes,^47–49^ our findings suggest a critical role of abnormal expression of IL-18 during COVID-19-associated cardiopulmonary damage. This notion is further reinforced by patient-level data where we observed higher levels of IL-18 in patients who died from COVID-19 compared to survivors. In fact, IL-18 was one of the top cytokines to discriminate mortality associated with COVID-19 among a survey of 19 cytokines after multi-variate adjustment. In addition, patients with higher IL-18 levels exhibited an increased risk of hospitalization burden after COVID-19 infection supporting similar observations about IL-18 in prior unadjusted studies.^5,50^ Notably, Rodrigues et al. found no significant difference in IL-18 levels between patients with severe versus mild/moderate COVID-19,^4^ highlighting the importance of IL-18 as a possible biomarker for late stages of COVID-19 rather than milder, early cases.

The ACE2/AngII/Ang-(1–7) axis has drawn much attention during the SARS-CoV-2 pandemic, due to its previously established role in regulating vasoconstriction, inflammation, and fibrosis.^51^ Prior work has demonstrated that reduced ACE2 expression levels mediated by Spike protein *alone* result in pulmonary vascular EC dysfunction and lung injury.^52^ Ang-(1–7) is known to ameliorate Spike protein-induced activation of the NLRP3 inflammasome.^40^ In the current work, Ang-(1–7) treatment inhibited IL-18 activation, supporting the notion that Ang II hyperactivation after ACE2 internalization during SARS-CoV-2 infection leads to excessive NLRP3 signaling and IL-18 activation.^51^

Defective mitophagy and the associated generation of ROS appear to closely correlate with an increased risk of developing cardiovascular and pulmonary vascular disorders.^53,54^ We observed evidence of damaged mitochondria and increased ROS that accumulates in the hearts of Spike protein-treated mice, supporting a role for mitoROS and mitophagy during COVID-19-related cardiac and pulmonary injury. In general, mitochondria disruption and the generation of mitoROS have been well-documented during inflammasome activation.^24^ The current study advances this link between selective mitophagy, Spike signaling, and IL-18 activation. Mechanistically, Spike protein treatment resulted in impaired mitophagy associated with ROS release, leading to IL-18 activation in both cardiomyocytes and lung ECs. Inhibition of ROS and augmenting mitophagy both reduced IL-18 expression despite exposure to Spike signaling. Additional studies are needed to determine whether mitophagy induction can alleviate IL-18-mediated cardiopulmonary injury.

This study also demonstrates that IL-15 levels are higher in blood and lung tissues from COVID-19 patients. IL-15 has been observed to play a synergistic effect with IL-18 in driving pathogen-induced NK cell proliferation,^55^ as well as antimicrobial and antitumoral protective immune responses.^56^ The interaction between IL-15 and IL-18 in COVID-19 pathogenesis remains to be characterized. In addition, pro-inflammatory cytokines (such as IL-1β, IL-6, C-reactive protein or TNF-α), proangiogenic factors (such as vascular endothelial growth factor, VEGF), vascular adhesion molecules (intercellular adhesion molecule-1, ICAM-1) and many others have been found to participate in pathogenesis of severe COVID-19,^4,57,58^ but were not analyzed in the current study. The potential confounding role of other, inflammatory cytokines and factors in COVD-19 outcomes as well as NLRP3 inflammasome activation and IL-18 production, not described in this work, are also important for study in future investigations.

In summary, IL-18 is a potential novel biomarker of disease severity and outcomes in patients with COVID-19. The hACE2 mouse model recapitulates patient-level findings, demonstrating increased IL-18 expression and cardiopulmonary injury after exposure to Spike protein. Our mouse work demonstrates that IL-18bp may represent a novel and targeted therapeutic for COVID-related cardiopulmonary inflammation and injury. Mechanisms of IL-18 activation appear to involve mitophagy blockade and ROS release, highlighting mitophagy and ROS as additional novel potential therapeutic targets for COVID-19-associated cardiopulmonary manifestations. Our observation that Spike protein alone can activate the inflammasome by inhibiting mitophagy may further have important implications with regard to vaccines that are based on administering Spike immunogens or inactive viruses that display Spike proteins.

## Materials and methods

### Indiana University (IU) COVID-19 cohort

The work was approved by the Institutional Review Board (IRB) at Indiana University (#1105005445). Informed consent was obtained by all subjects. Between December 31 and October 2, 2020, 109 patients hospitalized with suspicion of COVID-19 were tested and confirmed for SARS-CoV-2 viral infection status by PCR and prospectively underwent blood collection to profile cytokines. Samples for the RT–PCR SARS-CoV-2 lab test were collected via nasopharyngeal or oropharyngeal swab at different locations, representing outpatient, urgent care, emergency and inpatient facilities. Serum specimens for Bioplex screening were collected prospectively via venipuncture within Indiana University Health. De-identified clinical and demographic data were obtained from the Indiana Network for Patient Care (INPC) research database by Regenstrief Institute. Patient electronic health records were accessed for clinical data outcomes. Clinical follow-up data were collected up to October 2, 2020, for the cohort.

### Human lung samples

The patient was a 66-year-old man non-smoker with a history of hypertension. SARS-CoV-2 infection was identified by performing a real-time reverse transcriptase (RT)-PCR assay on a nasopharyngeal swab specimen and he was admitted to the hospital on 11 January 2020 with clinical symptoms of cough, fever, myalgia, and mild dyspnea. He developed respiratory failure and received lung transplantation on 25 February 2020. He died on 26 February 2020. More details of this case can be found in the following reference: Analysis of pathological changes in the epithelium in COVID-19 patient airways. Samples from the injured lungs of this patient were embedded in paraffin and sectioned for examination.

### Animal studies

All animal experiments were approved by the Ethics Committee of the First Affiliated Hospital of Guangzhou Medical University and were carried out according to University Guidelines for the Care and Use of Animals. C57BL/6J humanized ACE2 (hACE2) transgenic mice (ACE2-KI) and SARS-CoV-2 Spike RBD protein/S1 protein were respectively purchased from Cyagen Biotechnology (Guangzhou, China) and Sino Biological (Beijing, China). Adult mice (6–8 months old) of both sexes were administered recombinant SARS-CoV-2 Spike RBD protein or S1 protein (5 μg/mouse/d) for 10 days via tracheal intubation. Age and gender-matched control mice received equivalent doses of IgG-Fc protein. For IL-18BP treatment group, after 5 days of S1 protein administration, vehicle (PBS) or IL-18BP (0.5 mg/kg/day) were injected intraperitoneally in control and S1 mice for another 5 days. For urolithin A (UA, a mitophagy inducer) treatment group, vehicle (DMSO) or UA (25 mg/kg/day) were injected intraperitoneally in control and S1 mice for 10 days. For mitoquinone (MitoQ) treatment group, vehicle (DMSO) or MitoQ (5 mg/kg/day) were injected intraperitoneally in control and S1 mice once every other day for 10 days. All measurements and analyses were blinded.

### Statistics

All analyses and data processing were conducted using R version 4.0.4.^59^ IL-18 and other cytokine levels were obtained from the screening panel. For each patient, any values that were out of the range of the standard curve were excluded from analyses. IL-18 values were log transformed and classified into tertiles. Low tertile was defined as IL-18 ≤ 48.6 pg/mL, intermediate (“Mid”) tertile was defined as 102 pg/mL ≥ IL-18 ≥ 48.6 pg/mL, and high was IL-18 > 102 pg/mL. COVID-19 testing date was used for index date for time to outcome analysis. From the index date, time to last known discharge or death were used as censoring/event date. Hospitalization burden, the total numbers days spent hospitalized after coronavirus infection, was calculated as the total number of days hospitalized from all hospitalizations on or after COVID-19 test for each patient. Summary statistics for demographic, cytokine levels, and clinical variables were generated. For continuous variables, mean and standard deviation (sd) are shown. For categorical variables, the percentage and counts of samples with selected response level are shown. Univariate analysis of demographic, cytokine levels, and clinical variables were performed comparing those who lived versus those that died. Continuous variables were compared using Welch’s two sample test and categorical variables were compared using Fisher’s Exact test. Cox proportional hazard-based survival analysis of all-cause mortality was conducted with cytokine levels while adjusting for age, gender, and race using survival package^60^ in R. A *P* value less than 0.05 was defined as statistically significant. (∗, *P* values of ≤ 0.05. ∗∗, *P* values of ≤ 0.005. ∗∗∗, *P* values of ≤ 0.001.). For experimental work, ANOVA and Student’s t tests were also used to assess intergroup differences using GraphPad Prism. Data are presented as mean ± standard error of the mean (SEM). All the measurements and analyses were performed by scientists blinded to the treatment groups. Animals were randomly assigned to the respective groups.

Additional methods on TTE, ECG, and other protocols are available in the Supplemental Methods and Results.

## Supplementary information

Supplementary Materials for SARS-CoV-2 Spike Protein Induces IL-18-mediated Cardiopulmonary Inflammation Via Reduced Mitophagy

## Data Availability

All the data supporting the results of the present study are available from the corresponding authors upon reasonable request.

## References

[1] Agricola E (2020). Heart and lung multimodality imaging in COVID-19. JACC Cardiovasc. Imaging.

[2] Merad M, Martin JC (2020). Pathological inflammation in patients with COVID-19: a key role for monocytes and macrophages. Nat. Rev. Immunol..

[3] Turnquist C, Ryan BM, Horikawa I, Harris BT, Harris CC (2020). Cytokine storms in cancer and COVID-19. Cancer Cell.

[4] Rodrigues TS (2021). Inflammasomes are activated in response to SARS-CoV-2 infection and are associated with COVID-19 severity in patients. J. Exp. Med..

[5] Chi Y (2020). Serum cytokine and chemokine profile in relation to the severity of coronavirus disease 2019 in China. J. Infect. Dis..

[6] Lara PC, Macías-Verde D, Burgos-Burgos J (2020). Age-induced NLRP3 inflammasome over-activation increases lethality of SARS-CoV-2 pneumonia in elderly patients. Aging Dis..

[7] Van den Berg DF, Te Velde AA (2020). Severe COVID-19: NLRP3 inflammasome dysregulated. Front. Immunol..

[8] He Y, Hara H, Núñez G (2016). Mechanism and regulation of NLRP3 inflammasome activation. Trends Biochem. Sci..

[9] Dolinay T (2012). Inflammasome-regulated cytokines are critical mediators of acute lung injury. Am. J. Respir. Crit. Care Med..

[10] Huang KJ (2005). An interferon-gamma-related cytokine storm in SARS patients. J. Med. Virol..

[11] Jiang Y, Yi C, Yi Y, Jin Q, Kang AS (2020). Adiponectin exacerbates influenza infection in elderly individuals via IL-18. Signal. Transduct. Target Ther..

[12] Wu C (2020). Risk factors associated with acute respiratory distress syndrome and death in patients with coronavirus disease 2019 pneumonia in Wuhan, China. JAMA Intern. Med..

[13] Li S (2020). Clinical and pathological investigation of patients with severe COVID-19. JCI Insight.

[14] Barnes BJ (2020). Targeting potential drivers of COVID-19: neutrophil extracellular traps. J. Exp. Med..

[15] Tall AR, Westerterp M (2019). Inflammasomes, neutrophil extracellular traps, and cholesterol. J. Lipid Res..

[16] Pomerantz BJ, Reznikov LL, Harken AH, Dinarello CA (2001). Inhibition of caspase 1 reduces human myocardial ischemic dysfunction via inhibition of IL-18 and IL-1beta. Proc. Natl Acad. Sci. USA.

[17] Gupta A (2021). IL-18 mediates sickle cell cardiomyopathy and ventricular arrhythmias. Blood.

[18] Cascino TM, Desai AA, Kanthi Y (2021). At a crossroads: coronavirus disease 2019 recovery and the risk of pulmonary vascular disease. Curr. Opin. Pulm. Med..

[19] Chen Y (2021). Aging in COVID-19: vulnerability, immunity, and intervention. Ageing Res. Rev..

[20] Picca A, Guerra F, Calvani R (2019). Mitochondrial dysfunction and aging: insights from the analysis of extracellular vesicles. Int. J. Mol. Sci..

[21] Rea IM (2018). Age and age-related diseases: role of inflammation triggers and cytokines. Front. Immunol..

[22] Zhou R, Yazdi AS, Menu P, Tschopp J (2011). A role for mitochondria in NLRP3 inflammasome activation. Nature..

[23] Pickles S, Vigié P, Youle RJ (2018). Mitophagy and quality control mechanisms in mitochondrial maintenance. Curr. Biol..

[24] Yuk JM, Silwal P, Jo EK (2020). Inflammasome and mitophagy connection in health and disease. Int. J. Mol. Sci..

[25] Potus F, Mai V, Lebret M, Malenfant S (2020). Novel insights on the pulmonary vascular consequences of COVID-19. Am. J. Physiol. Lung Cell Mol. Physiol..

[26] Lupfer C (2013). Receptor interacting protein kinase 2-mediated mitophagy regulates inflammasome activation during virus infection. Nat. Immunol..

[27] Galván-Román JM (2021). IL-6 serum levels predict severity and response to tocilizumab in COVID-19: An observational study. J. Allergy Clin. Immunol..

[28] Walls AC (2020). Structure, function, and antigenicity of the SARS-CoV-2 spike glycoprotein. Cell..

[29] Zhou F (2020). Clinical course and risk factors for mortality of adult inpatients with COVID-19 in Wuhan, China: a retrospective cohort study. Lancet..

[30] Schroder K, Tschopp J (2010). The inflammasomes. Cell..

[31] Wang P (2020). A cross-talk between epithelium and endothelium mediates human alveolar-capillary injury during SARS-CoV-2 infection. Cell Death Dis..

[32] Gibellini L, De Biasi S (2020). Altered bioenergetics and mitochondrial dysfunction of monocytes in patients with COVID-19 pneumonia. EMBO Mol. Med..

[33] Bojkova D (2020). SARS-CoV-2 infects and induces cytotoxic effects in human cardiomyocytes. Cardiovasc. Res..

[34] Chen L, Li X, Chen M, Feng Y, Xiong C (2020). The ACE2 expression in human heart indicates new potential mechanism of heart injury among patients infected with SARS-CoV-2. Cardiovasc. Res..

[35] Colunga Biancatelli RML, Solopov PA (2021). The SARS-CoV-2 spike protein subunit S1 induces COVID-19-like acute lung injury in Κ18-hACE2 transgenic mice and barrier dysfunction in human endothelial cells. Am. J. Physiol. Lung Cell. Mol. Physiol..

[36] Palikaras K, Lionaki E, Tavernarakis N (2018). Mechanisms of mitophagy in cellular homeostasis, physiology, and pathology. Nat Cell Biol..

[37] Evans PC (2020). Endothelial dysfunction in COVID-19: a position paper of the ESC Working Group for Atherosclerosis and Vascular Biology, and the ESC Council of Basic Cardiovascular Science. Cardiovasc. Res..

[38] Barbosa LC, Gonçalves TL, de Araujo LP, Rosario LVO, Ferrer VP (2021). Endothelial cells and SARS-CoV-2: an intimate relationship. Vascul. Pharmacol..

[39] Libby P, Lüscher T (2020). COVID-19 is, in the end, an endothelial disease. Eur. Heart J..

[40] Ratajczak MZ (2021). SARS-CoV-2 entry receptor ACE2 is expressed on very small CD45(-) precursors of hematopoietic and endothelial cells and in response to virus spike protein activates the Nlrp3 inflammasome. Stem Cell Rev. Rep..

[41] Pons S, Fodil S, Azoulay E, Zafrani L (2020). The vascular endothelium: the cornerstone of organ dysfunction in severe SARS-CoV-2 infection. Crit Care..

[42] Gu T (2020). Cytokine signature induced by SARS-CoV-2 spike protein in a Mouse Model. Front. Immunol..

[43] Dinnon KH, Leist SR, Schäfer A, Edwards CE (2020). A mouse-adapted model of SARS-CoV-2 to test COVID-19 countermeasures. Nature.

[44] Paidi RK (2021). ACE-2-interacting domain of SARS-CoV-2 (AIDS) peptide suppresses inflammation to reduce fever and protect lungs and heart in mice: implications for COVID-19 therapy. J. Neuroimmune Pharmacol..

[45] Clerkin KJ (2020). COVID-19 and cardiovascular disease. Circulation.

[46] Vecchié A, Bonaventura A (2021). IL-18 and infections: is there a role for targeted therapies?. J. Cell Physiol..

[47] Driver TH (2014). Urinary kidney injury molecule 1 (KIM-1) and interleukin 18 (IL-18) as risk markers for heart failure in older adults: the Health, Aging, and Body Composition (Health ABC) Study. Am. J. Kidney Dis..

[48] Abbate A (2020). Interleukin-1 and the inflammasome as therapeutic targets in cardiovascular disease. Circ Res..

[49] Tong Y, Wang Z, Cai L, Lin L, Liu J (2020). NLRP3 inflammasome and its central role in the cardiovascular diseases. Oxid. Med. Cell Longev..

[50] Satış H (2021). Prognostic value of interleukin-18 and its association with other inflammatory markers and disease severity in COVID-19. Cytokine.

[51] Mori J, Oudit GY, Lopaschuk GD (2020). SARS-CoV-2 perturbs the renin-angiotensin system and energy metabolism. Am. J. Physiol. Endocrinol. Metab..

[52] Lei Y (2021). SARS-CoV-2 spike protein impairs endothelial function via downregulation of ACE 2. Circ Res..

[53] Bravo-San Pedro JM, Kroemer G, Galluzzi L (2017). Autophagy and mitophagy in cardiovascular disease. Circ Res..

[54] Morales PE (2020). Emerging role of mitophagy in cardiovascular physiology and pathology. Mol. Aspects Med..

[55] French AR, Holroyd EB, Yang L, Kim S, Yokoyama WM (2006). IL-18 acts synergistically with IL-15 in stimulating natural killer cell proliferation. Cytokine.

[56] Soudja SM, Ruiz AL, Marie JC, Lauvau G (2012). Inflammatory monocytes activate memory CD8(+) T and innate NK lymphocytes independent of cognate antigen during microbial pathogen invasion. Immunity.

[57] Smadja DM (2021). COVID-19 is a systemic vascular hemopathy: insight for mechanistic and clinical aspects. Angiogenesis.

[58] Del Valle DM, Kim-Schulze S, Huang HH, Beckmann ND, Nirenberg S (2020). An inflammatory cytokine signature predicts COVID-19 severity and survival. Nat. Med..

[59] Team, R. C. *R: A Language and Environment for Statistical Computing* (R Foundation for Statistical Computing, Vienna, Austria, 2019).

[60] Therneau, T. *A Package for Survival Analysis in R*. R package version 3.5-3. https://CRAN.R-project.org/package=survival (2023).

