# Supplementary material for: SARS-CoV-2 spike protein induces IL-18-mediated cardiopulmonary inflammation via reduced mitophagy
Source: Signal Transduct Target Ther. 2023 Mar 9;8:108. doi: 10.1038/s41392-023-01368-w (PMC9998025; doi:10.1038/s41392-023-01368-w)
Supplement: Supplementary file 1 — Supplementary Materials for SARS-CoV-2 Spike Protein Induces IL-18-mediated Cardiopulmonary Inflammation Via Reduced Mitophagy [file 41392_2023_1368_MOESM1_ESM.docx]

Supplementary Materials for

SARS-CoV-2 Spike Protein Induces IL-18-mediated Cardiopulmonary Inflammation Via Reduced Mitophagy

Shuxin Liang^1†^, Changlei Bao^1†^, Zi Yang^1^, Shiyun Liu^1^, Yanan Sun^1,2^, Weitao Cao^1^, Ting Wang^3,4^, Tae-Hwi Schwantes-An^5^, John Choy^6^, Samisubbu Naidu^7^, Ang Luo^1,2^, Wenguang Yin^1^, Stephen M. Black^3,4^, Jian Wang^1^, Pixin Ran^1,8^, Ankit A. Desai^7*^, Haiyang Tang^1*^

Correspondence to: Haiyang Tang (tanghy2008@yahoo.com); Ankit A. Desai (ankdesai@iu.edu)

**This PDF file includes:**

Materials and Methods

Figures. S1 to S6

**SUPPLEMENTAL METHODS**

**Human Lung Samples**. The patient was a 66-year-old man non-smoker with the comorbidity of hypertension. SARS-CoV-2 infection was identified by performing a real-time reverse transcriptase (RT)-PCR assay on a nasopharyngeal swab specimen and he was admitted to the hospital on 11 January 2020 with clinical symptoms of cough, fever, myalgia and mild dyspnoea. He developed respiratory failure, received lung transplantation therapy on 25 February 2020, and was declared clinically dead on 26 February 2020. More details refer to this literature (Analysis of pathological changes in the epithelium in COVID-19 patient airways). Samples from the injured lungs of this patient were embedded in paraffin and sectioned for examination.

**Cytokine Screening Panel**. The Bio-Plex Pro Human Cytokine Screening Panel, 48-Plex (#12007283) platform is a rapid cytokine detection system based on ELISA assays run in triplicate following the manufacturer’s instructions. Analytical validation was performed using both reference cytokine controls and biological replicates across different lots.

**Animal studies**. All animal experiments were approved by the Ethics Committee of the First Affiliated Hospital of Guangzhou Medical University and were carried out according to University Guidelines for the Care and Use of Animals. C57BL/6J humanized ACE2 (hACE2) transgenic mice (ACE2-KI) and SARS-CoV-2 Spike RBD protein/S1 protein were respectively purchased from Cyagen Biotechnology (Guangzhou, China) and Sino Biological (Beijing, China). Adult mice (6–8 months old) of both sexes were administered recombinant SARS-CoV-2 Spike RBD protein or S1 protein (5 μg/mouse/d) for 10 days via tracheal intubation. Age and gender-matched control mice received equivalent doses of IgG-Fc protein. For IL-18BP treatment group, after 5 days of S1 protein administration, vehicle (PBS) or IL-18BP (0.5 mg/kg/day) were injected intraperitoneally in control and S1 mice for another 5 days. For urolithin A (UA, a mitophagy inducer) treatment group, vehicle (DMSO) or UA (25 mg/kg/day) were injected intraperitoneally in control and S1 mice for 10 days. For mitoquinone (MitoQ) treatment group, vehicle (DMSO) or MitoQ (25 mg/kg/day) were injected intraperitoneally in control and S1 mice once every other day for 10 days. All measurements and analyses were blinded.

**Cell culture**. HPAEC and HCMEC were maintained in fibronectin (ScienCell, 8248)-coated culture vessels with EC medium (ScienCell, 1001), cultured in a 37°C, 5% CO_2_, humidified incubator and used between passages 4 and 5. H9C2 cell lines were maintained in DMEM medium (Gibco, 11965092) supplemented with 10% FBS (Gibco, 10099141) and 1% penicillin/streptomycin (Gibco, 15140122).

**Establishment of a stable human ACE2 expressing cell line**. The lentivirus FLAG-hACE2/FLAG-GV358 were provided by Genechem Co. Ltd (Shanghai，China). H9C2 cells were transduced by lentivirus (multiplicity of infection, MOI = 10) at 60-70% confluence with polybrene (8 μg/ml). After 48-72 h post-transduction, puromycin (8 μg/mL, Sigma-Aldrich, P8833) was added to screen hACE2 expressing cell lines and then puromycin-resistant clones were sorted and checked by western blot.

**Flow cytometry analysis**. For cell binding and internalization assay, cells were cultured with 2.5 μg/mL S1-Fc, RBD-Fc or Fc protein (Sino bioloigcal) for 3 hours, respectively. Then, cells were washed with cold PBS and labeled with a FITC-conjugated goat anti-human IgG (H + L) antibody (1: 100, Invitrogen, A18818) at 4 °C for 2 hours in the dark. After washings with cold PBS for three times, the cells were resuspended in PBS for flow cytometry.

For mitoROS measurements, assays were performed according to the manufacturer’s instructions (Invitrogen, M36008). In brief, cells were washed with PBS and then incubated with MitoSOX™ Red (5 μM for 30 min) in the dark. Fluorescence was detected with flow cytometry. All flow cytometry data were obtained using a BD FACSVerse and were analyzed using FlowJo software.

**Endothelial cell permeability assays**. Endothelial cell permeability was evaluated by an *in vitro* vascular permeability assay (Sigma-Aldrich, ECM644) following the manufacturer’s instructions. In brief, cells were seeded in the insert for 36**–**72 hours until a monolayer is formed and starved for 6**–**12 hours before adding RBD proteins with different concentration, S1 proteins (5 μg/mL) or IL-18BP (2 μg/mL). A 150 µL of FITC-Dextran working solution (1:40) was applied and incubated for 20 mins in the dark at room temperature. 100 µL of the media in lower chamber was transferred into opaque plate for fluorescence measurements. Fluorescence was measured in 485 nm and 535 nm excitation and emission, respectively.

**Transmission electron microscopy (TEM)**. Collected cells were fixed with TEM fixative (Servicebio, G1102). Samples were washed with PBS and wrapped in the agarose. Then, Samples were post fixed in 1% osmium tetroxide in 0.1 M phosphate buffer (pH 7.4) for 2 hours in the dark and dehydrated by a graded series of ethanol, followed by penetration and polymerization in EMBed 812 resins. Ultrathin section (60**–**80 nm thin) was produced by ultramicrotome and stained with 2% uranium acetate saturated alcohol solution, followed by 2.6% lead citrate. Samples are taken images under TEM.

**Transthoracic echocardiography (TTE)****.** We used Vevo 2100 (FUJIFILM VisualSonics Inc, Toronto, Canada) to evaluate cardiac structure and function at baseline and 10 days after treatment. Prior to TTE studies, mice were anesthetized with 1–1.5% isoflurane and chest hair were removed. Following published protocols (REFS), we evaluated left ventricular chamber size and systolic function via 4-chamber, 2-chamber, and parasternal long, and short axis views. M-mode images of the left ventricle (LV) in the parasternal long axis (PSLAX) and short-axis (SAX) views at the mid-papillary level were taken to assess wall thickness and mass. The following variables were also collected or calculated: heart rate (HR), end diastolic (EDV) and systolic (ESV) LV chamber volumes and wall thickness (spetal and laterall walls), ejection fraction (EF), fractional shortening (FS), stroke volume (SV), and cardiac output (CO). To evaluate LV diastolic function, mitral pulse wave Doppler flow and tissue Doppler imaging (TDI) in the apical four-chamber view were performed. Mitral inflow velocity, peak early (E) and late (A) were measured by conventional pulsed-wave Doppler. TDI was used to determine the mitral annulus longitudinal velocity, early (E’) and late (A’).

**Non-invasive electrocardiograms (ECG) recording.** A standard Ⅱ lead ECG of mice under anesthesia with 1–1.5% isoflurane was recorded directly with a subcutaneous electrode. Labchart Pro 8.0 (Power Lab 4/35 Model) was used for the original data storage and analysis. ECG parameters including PR, RR, QRS, QT, and QTc (corrected) intervals, P wave amplitude and duration, Tpeak-Tend (Tp-Te) interval, and ST segment changes were measured.

**Histological study.** After treatment, animals were anesthetized with 2% isoflurane followed by transcardial perfusion. The hearts and lungs were collected and processed for histological studies. Tissues samples were fixed in 10% Formalin solution, embedded in paraffin and 4 μm thick sections were used for histology. To detect lung tissue morphology, hematoxylin and eosin (HE) staining was performed. For number of infiltrated neutrophils quantification, at least fifteen 40× fields from each group were selected for the counting of the infiltrated neutrophils. Lung injury score was analyzed according to the Matute-Bello et al. [[22](#_ENREF_22)]. Masson’s Trichrome staining was performed for identification of collagen. Heart fibrosis was assessed by determination of collagen density on tissues obtained from the cross-sections. Collagen volume density fraction was determined as the sum of stained collagen tissue divided by the sum of all stained muscle and connective tissue in the visual field using Image J software. At least ten 50× fields were randomly selected for each heart.

**Immunofluorescence** **and confocal microscopy.** Cells were seeded in coverslips with the relevant treatment. After washing with PBS, fixed with 4% paraformaldehyde in PBS, permeabilized with 0.1% Triton X-100, and blocked with 10% BSA, cells were incubated with primary antibody at 4°C overnight, then incubated with Alexa Fluor 488 and 596-conjugated secondary antibodies for 2 hrs at room temperature followed by the counterstain of nuclei with DAPI. Primary antibody against TOM20 (1:200 dilution, 42406), LAMP1 (1:200 dilution, 9091) and LC3B (1:200 dilution, 83506) were obtained from Cell Signaling Technology. Images were visualized using a confocal microscope (Carl Zeiss Microimaging Inc, Welwyn Garden City, UK).

**Western blot and quantitative RT-PCR analysis.** Protein samples were extracted using RIPA buffer (Pierce, Rockford, IL, USA). Identical amounts of protein were separated by SDS-PAGE and then transferred to PVDF membranes (Millipore, ISEQ00010). Membranes were blocked via 10% skim milk in TBST and incubated with appropriate primary antibodies and Secondary antibodies. Anti-IL-18 (ab191860; ab191152) and Anti-α-Tubulin (ab7291) were from Abcam. Anti-ASC (13833), Anti-IκBα (9242), Anti-NF-κB p65 (8242), Anti-phospho-NF-κB p65 (3033) and anti-TOM20 (42406) were purchased from Cell Signaling Technology. Anti-LC3B (L7543) and anti-FLAG (F1804) were from Sigma-Aldrich. Anti-ASC (NBP1-78977), Anti-NLRP3 (NBP2-12446), Anti-NOX4 (NB110-58851) and Anti-ACE2 (NBP1-76614) was from Novus Biologicals. Anti-β-actin (60008-1-Ig) was from Proteintech. Secondary antibodies (7076 and 7074) were purchased from Cell Signaling Technology. proteins were detected using a SuperSignal West Pico Kit (Thermo Fisher Scientific, 34578).

RNA extraction was carried out using the Trizol reagent (Invitrogen, 93289) according to the manufacturer’s instructions. RT-qPCR was performed using the TB Green Premix Ex Taq II (TaKaRa, RR82LR) with specific primers on the Bio-Rad CFX96. Primer sequences used in the study are: *Il-18* forward: TACAAGCATCCAGGCACAGC, *Il-18* reverse: CTGATGCTGGAGGTTGCAGA. The relative changes in gene expression were calculated using 2^-ΔΔCt^ method, normalized to β-actin.

**SUPPLEMENTAL FIGURES**


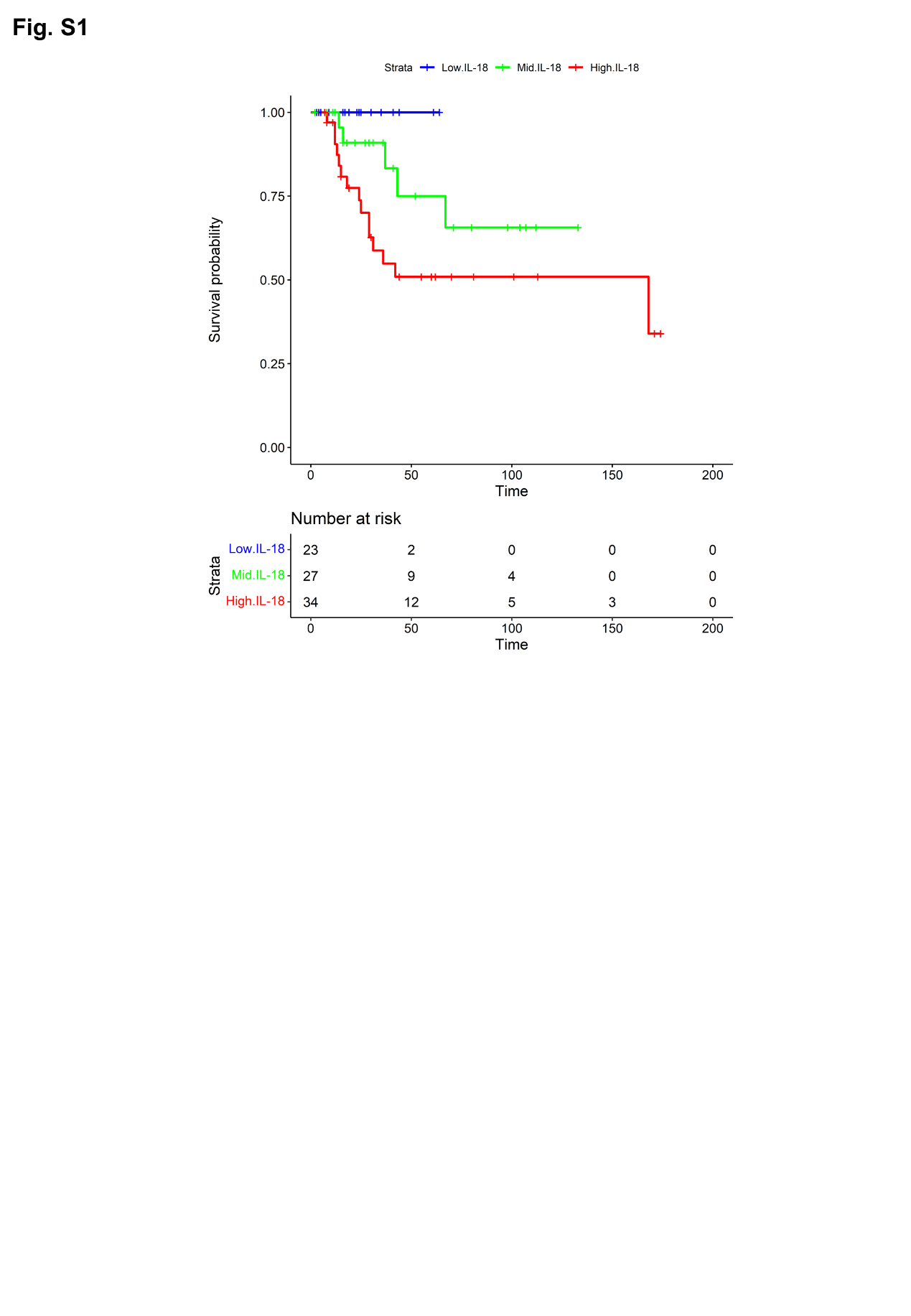


**Figure S1. Survival curves stratified by IL-18 levels after COVID-19 diagnosis.** Kaplan-Meier curves illustrate significant overall survival differences based on IL-18 tertiles based on time from COVID-19 test to last follow-up.


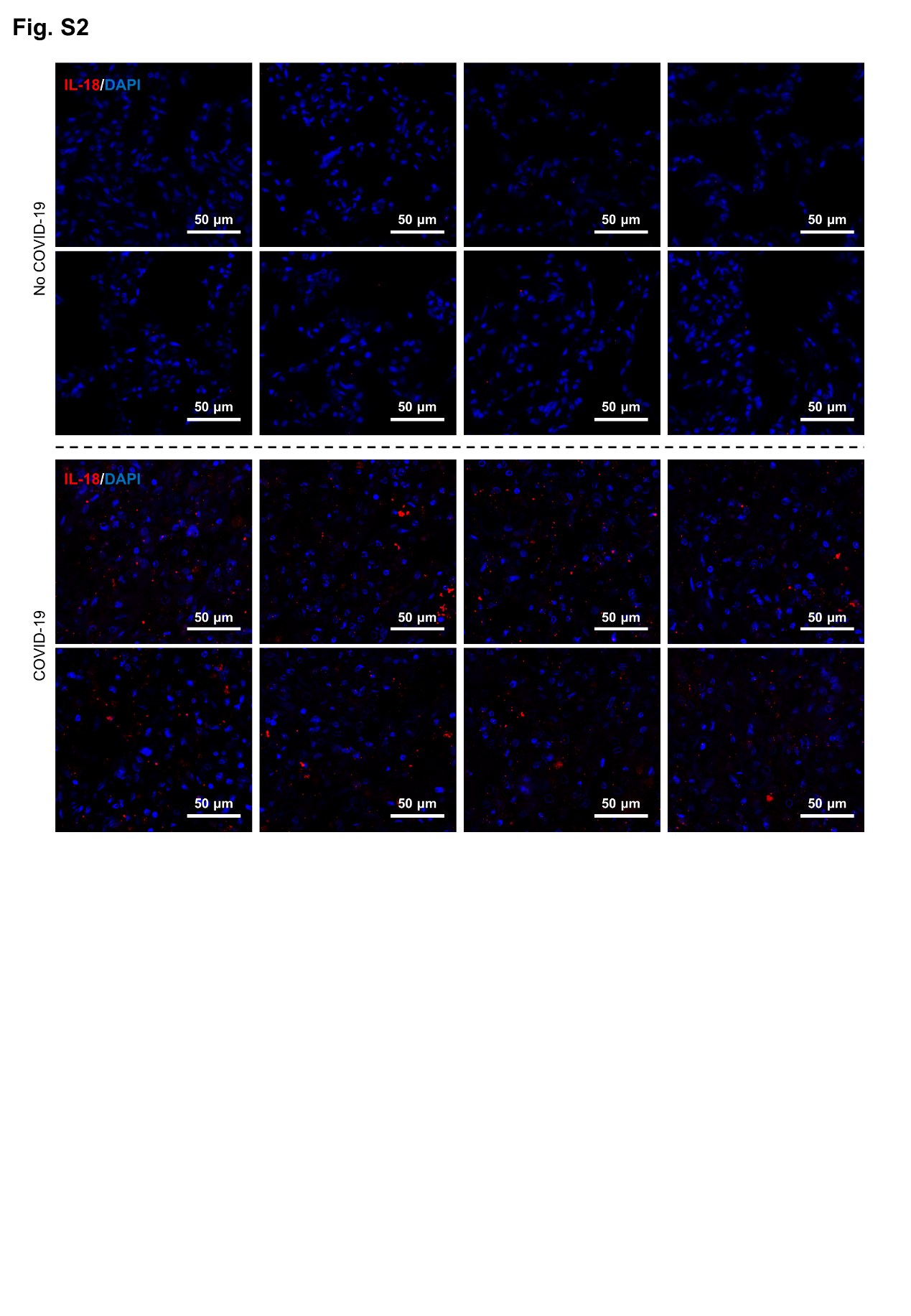


**Figure S2. Raw data for IL-18 staining in the lung tissues from no COVID-19 and COVID-19 patients.** Scale bar, 50 μm.


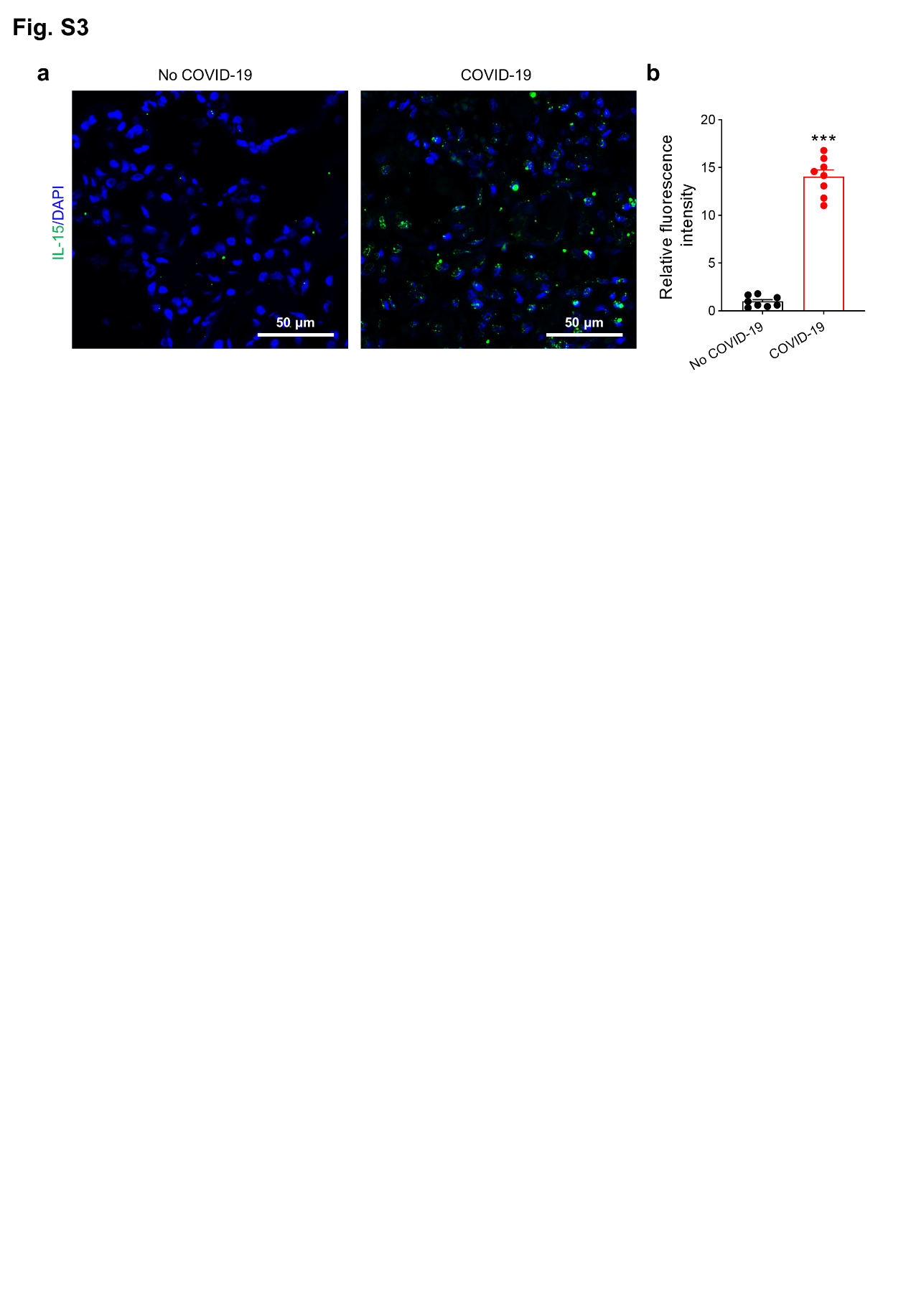


**Figure S3. IL-15 expression increases in human lung from COVID-19 patients.** Representative immunofluorescence images **(a)** and quantitative data **(b)** showing stained IL-15 in human lung from no COVID-19 and COVID-19 patients (Student’s *t*-test). Nuclei counterstained with DAPI. Scale bar, 50 μm. Values are mean ± SE. ***p < 0.001.


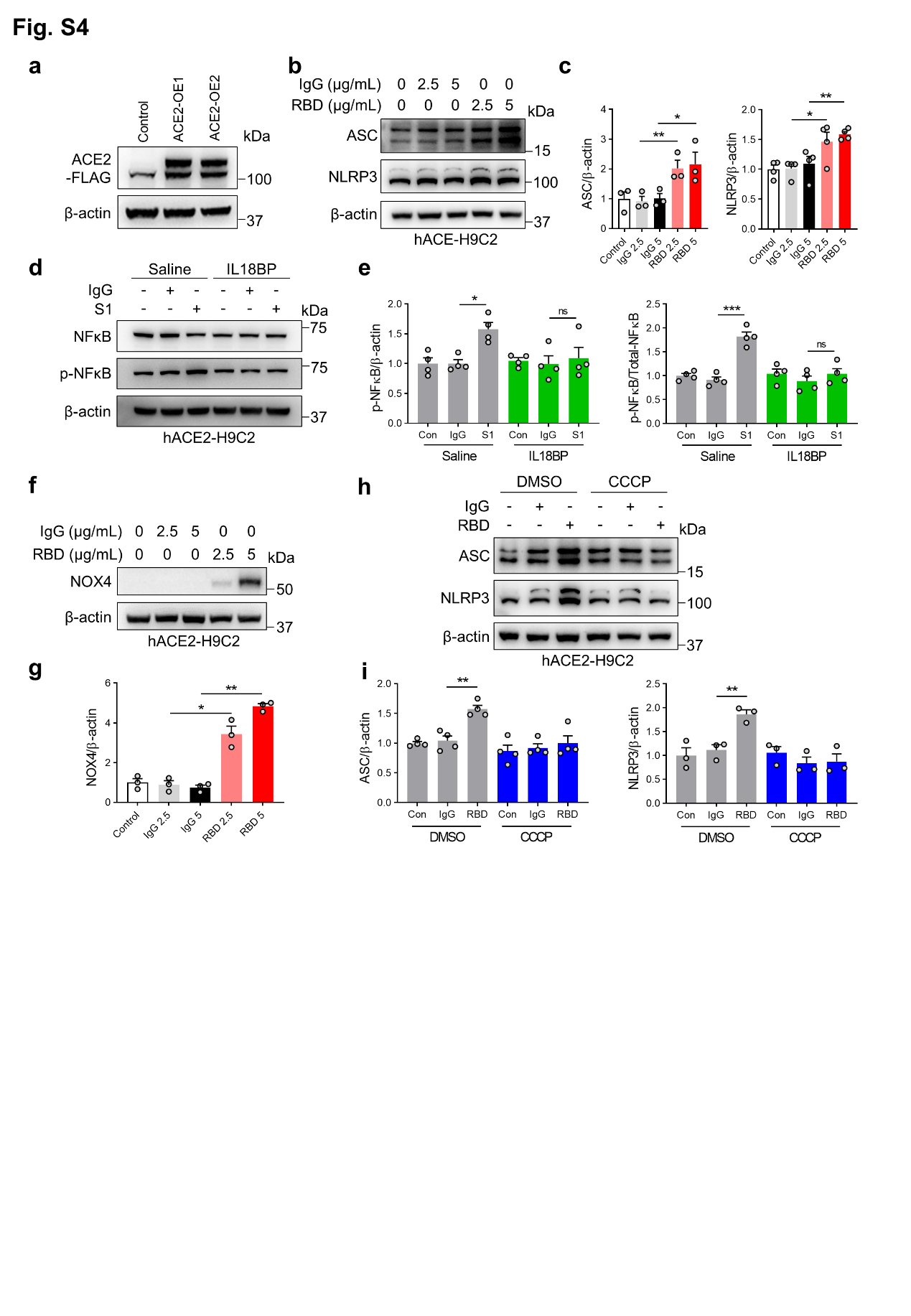


**Figure S4.** **Spike RBD protein activates NLRP3 inflammasome via inhibiting mitophagy in hACE2-H9C2 cells. (a)** hACE2-H9C2 cells were analyzed by western blot for expressed ACE2. **(b and c)** ASC and NLRP3 protein levels were measured using western blot (Student’s *t*-test，N=3-4). **(d and e)** hACE2-H9C2 cells were pretreated with IL-18BP (2 μg/mL) for 1 hour and then treated with IgG or S1 protein (2.5 μg/mL). NF-κB and phospho-NF-κB protein levels were measured using western blot (1-way ANOVA test, N=4). **(f and g)** Representative western blot images showing protein level of NOX4 (Student’s *t*-test, N=3). **(h and i)** Western blot analysis of ASC and NLRP3 in the presence of IgG or RBD protein (2.5 μg/mL) and CCCP (10 μM) (1-way ANOVA test, N=3). Values are mean ± SE. *p < 0.05, **p < 0.01.


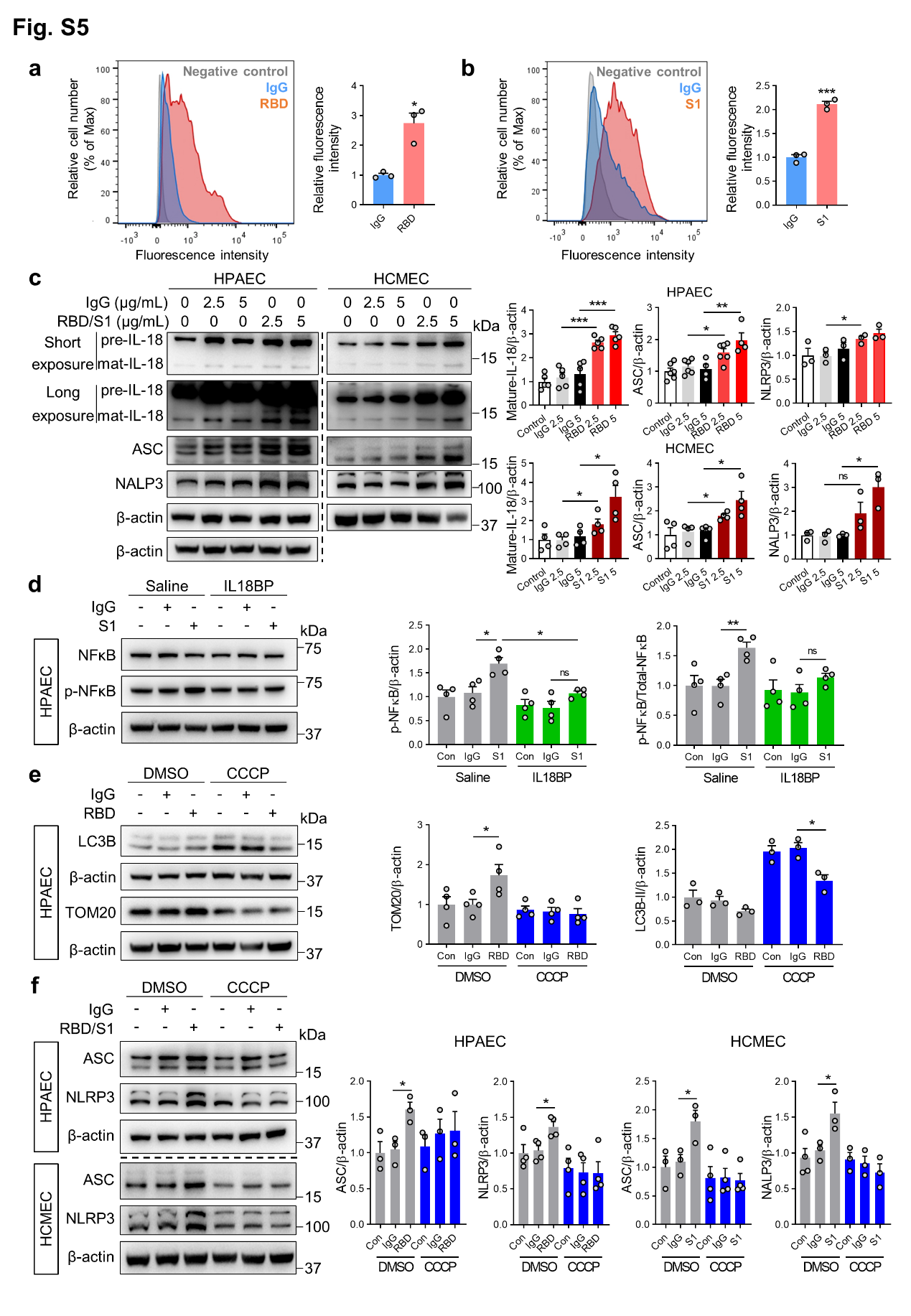


**Figure S5. Spike protein induces** **NLRP3 and ASC expression via reduction in mitophagy in HPAEC and HCMEC.** **(a)** Representative FACS histograms showing the binding of RBD protein to HPAEC surface. (Student’s *t*-test，N=3). **(b)** Representative FACS histograms showing the binding of S1 protein to HCMEC surface. (Student’s *t*-test，N=3) **(c)** Immunoblot analysis of IL-18, NLRP3 and ASC after treatment with Spike protein in EC (Student’s *t*-test, N=3-6). **(d)** HPAEC were pretreated with IL-18BP (2 μg/mL) for 1 hour and then stimulated with IgG or S1 protein (2.5 μg/mL). Phosphorylation of NF-κB was measured by western blotting (1-way ANOVA test, N=4). **(e)** LC3B and TOM20 expression was assessed under CCCP (10 μM) and RBD protein (2.5 μg/mL) treatment by immunoblots analysis. Representative results and summary data are presented (1-way ANOVA test, N=3-4). **(f)** Protein extracts were subjected to western blot analysis for NLRP3 and ASC after CCCP ( 10 μM) and RBD protein (2.5 μg/mL) treatment in EC (1-way ANOVA test, N=3-4). Values are mean ± SE. *p < 0.05, **p < 0.01, ***p < 0.001.

**
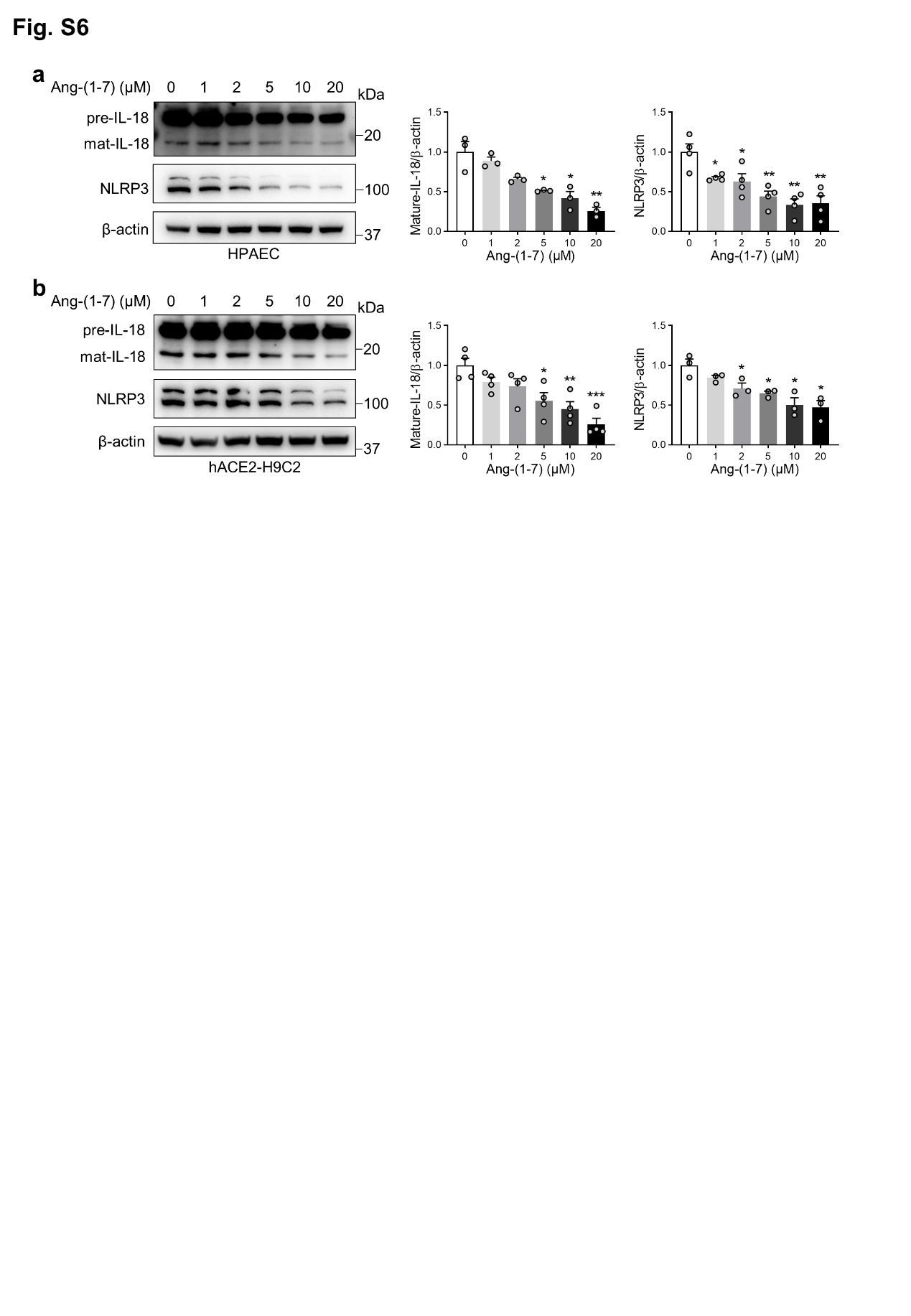
**

**Figure S6. Ang-(1-7) inhibit IL-18 expression. (A and B)** IL-18 and NLRP3 expression after 48h exposure to Ang-(1-7) in HPAEC **(A)** and hACE2-H9C2 cells **(B)**. Representative western blot results are presented (Student’s *t*-test, N=3-4). Results represent means ± SE. *p < 0.05, **p < 0.01, ***p < 0.001.
